# Controllable Thin‐Film Approaches for Doping and Alloying Transition Metal Dichalcogenides Monolayers

**DOI:** 10.1002/advs.202004249

**Published:** 2021-02-26

**Authors:** Yu‐Chuan Lin, Riccardo Torsi, David B. Geohegan, Joshua A. Robinson, Kai Xiao

**Affiliations:** ^1^ Department of Materials Science and Engineering The Pennsylvania State University University Park PA 16802 USA; ^2^ Center for Nanophase Materials Sciences Oak Ridge National Laboratory Oak Ridge TN 37831 USA; ^3^ Two‐Dimensional Crystal Consortium The Pennsylvania State University University Park PA 16802 USA; ^4^ Center for 2‐Dimensional and Layered Materials The Pennsylvania State University University Park PA 16802 USA

**Keywords:** 2D materials, alloy, doping, implantation, laser, thin‐film techniques, transition metal dichalcogenides

## Abstract

Two‐dimensional (2D) transition metal dichalcogenides (TMDs) exhibit exciting properties and versatile material chemistry that are promising for device miniaturization, energy, quantum information science, and optoelectronics. Their outstanding structural stability permits the introduction of various foreign dopants that can modulate their optical and electronic properties and induce phase transitions, thereby adding new functionalities such as magnetism, ferroelectricity, and quantum states. To accelerate their technological readiness, it is essential to develop controllable synthesis and processing techniques to precisely engineer the compositions and phases of 2D TMDs. While most reviews emphasize properties and applications of doped TMDs, here, recent progress on thin‐film synthesis and processing techniques that show excellent controllability for substitutional doping of 2D TMDs are reported. These techniques are categorized into bottom–up methods that grow doped samples on substrates directly and top–down methods that use energetic sources to implant dopants into existing 2D crystals. The doped and alloyed variants from Group VI TMDs will be at the center of technical discussions, as they are expected to play essential roles in next‐generation optoelectronic applications. Theoretical backgrounds based on first principles calculations will precede the technical discussions to help the reader understand each element's likelihood of substitutional doping and the expected impact on the material properties.

## Graphical abstract

Doping and alloying two‐dimensional (2D) materials offer tremendous opportunities for tuning their properties and adding new functionalities. In this review, recent progress on the non‐equilibrium thin‐film techniques that can provide scalable, large‐area synthesis and good degrees of controllability for the compositional engineering of 2D materials are summarized, especially focusing on semiconducting transition metal dichalcogenides including methods from bottom–up synthesis to top–down processing.

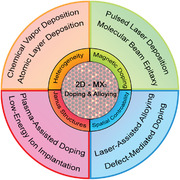

## 1 Introduction

**Figure 1:**
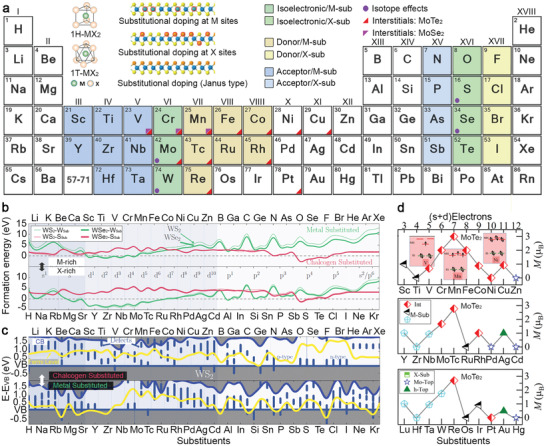
Theoretical overview of the potential dopants for group‐VI 2D TMDs. a) DFT calculations highlight the elements that can substitute the transition metal element sites (M) or chalcogen element sites (X) of 1H‐ group‐VI TMDs with the formation energy (*E*_f_) less than 1 eV or negative *E*_f_. Based on their role as either donor (p‐doping) or acceptor (n‐doping), they are colored with blue and yellow in different color tones. In addition to substitutional doping, dopants that are predicted to be stable in the interstitials of MoSe_2_ and MoTe_2_ by DFT calculations are marked with colored triangles. Dopants that can provide isotope effects and have been reported recently are marked with a purple circle. Inset provides the unit cell of 1H‐ and 1T‐MX_2_ monolayers. Reproduced with permission.^[^ ^6^ ^]^ Copyright 2015, American Chemical Society; Schematic side view (lower panel) of the crystal structure of substitutionally doped monolayer TMD at M sites and X sites and the Janus structures with only the top‐most X sites replaced. Reproduced with permission.^[^ ^8^ ^]^ Copyright 2017, Wiley‐VCH. b) The formation energy of W‐substituted (green) and chalcogen‐substituted (red) WX_2_ (with X = S, Se) in M‐rich and X‐rich conditions as a function of the substituent. Reproduced with permission.^[^ ^32^ ^]^ Copyright 2017, AIP Publishing. c) The energy of the conduction band (CB, navy blue), Fermi level (yellow), and defects in the bandgap (blue) with respect to the energy of the valence band (VB, blue line *x* = 0) of S‐ (top) and W‐(bottom) substituted monolayer WS_2_ as a function of substituents. Reproduced with permission.^[^ ^32^ ^]^ Copyright 2017, AIP Publishing. d) Panel shows magnetic moments (*M*) of the lowest energy configurations of transition metal substituents in MoTe_2_ layers. The inset panel presents a schematic of the d orbital splitting for interstitial TM atoms. Reproduced with permission.^[^ ^37^ ^]^ Copyright 2019, American Chemical Society.

The family of atomically‐thin two‐dimensional (2D) transition metal dichalcogenides (TMDs) has indisputably advanced fundamental solid‐state physics of layered materials and has become a mainstay of nanotechnology due to their ultra‐thin nature and confinement effects.^[^ ^1^ ^]^ The demonstration of high‐performance monolayer MoS_2_ field‐effect transistors (FET) in 2011^[^ ^2^ ^]^ shifted the focus in 2D materials from graphene to the TMD family.^[^ ^3^ ^]^ While graphene is only one carbon atom thick and provides useful properties from its Dirac‐cone band structure, its gapless nature limits many optoelectronic applications to only within the range of infrared wavelengths. Many efforts for opening a bandgap in graphene have been devoted to straining graphene or creating nanoribbons with 4–8 nm in width.^[^ ^4^, ^5^ ^]^ However, the bandgap induced in graphene by these methods is only hundreds of meV; thus, it is not enough for most applications in the range of visible wavelengths.^[^ ^5^ ^]^ Generally, 2D TMDs have a generalized formula MX_2_ comprised of two outer atomic layers of chalcogens that sandwich an atomic layer of transition metals, making a monolayer 0.6–0.7 nm in thickness. Their intralayer M—X bonds are covalent, whereas their two surfaces are dangling bond‐free and exhibit weak vdW forces.^[^ ^6^ ^]^ The transition metal coordination of thermodynamically stable TMD monolayers can be either trigonal prismatic (1H) or octahedral (1T) phase (**Figure** **1a**), depending on the combination of the metal and chalcogen constituents. The band structure of a TMD monolayer is dictated by the crystal phase and the d‐orbital electron numbers in its transition metal constituent. When the orbitals are only partially filled, TMDs are metallic. When the orbitals are filled, on the other hand, TMDs are semiconducting. The d‐orbital electron numbers of the transition metal elements on the periodic table also dictate the most stable crystal phase. For example, Group V TMD with d^0^ transition metal (Ti, Zr) at the coordination center are in 1T phase, whereas Group VI TMD with d^2^ metals (Mo, W) are in 1H phase.^[^ ^1^, ^6^ ^]^

Many 2D materials demonstrate novel functionalities by doping the materials or forming ternary and quaternary 2D alloys via deliberate inclusion of heteroatoms.^[^ ^7^, ^8^ ^]^ Doping through surface functionalization with molecular donors/acceptors or transition metal oxides are popular options because they can be conducted at room temperature and are effective on a material with a high surface‐to‐volume ratio. However, robust, long‐term doping requires a substitutional process that secures functional elements into their lattice. If 2D TMDs from each transition metal group could accept many types of dopants on the periodic table and remain thermodynamically stable, a tremendous number of new functionalities could be created. For example, semiconducting 2D TMDs made of Group VI transition metals (Mo and W) and Group XVI chalcogens (S, Se, and Te) are substitutionally doped Group V (e.g., Nb^[^ ^9^ ^]^) and Group VII (e.g., Re^[^ ^10^ ^]^) elements for p‐type and n‐type doping, respectively. Similarly, the S, Se, and Te constituents could be substituted with Group XV (e.g., N^[^ ^11^ ^]^ and P^[^ ^12^, ^13^ ^]^) and Group XVII (e.g., F and Cl) elements for p‐ and n‐doping, respectively. TMDs consisting of heavier constituents of Group XVI (e.g., S to Te) have a smaller bandgap. Magnetic dopants, including Fe,^[^ ^14^ ^]^ Co,^[^ ^15^ ^]^ and Mn^[^ ^16^, ^17^ ^]^ can potentially introduce ferromagnetism into non‐magnetic 2D TMDs. Additionally, their electronic structures and crystal phases become tunable when adequate amounts of substituents turn a TMD into an alloy and start to impact the lattice constant and thermal stability. In addition to tuning the bandgap sizes,^[^ ^18^ ^]^ alloying can tune the optical, electronic and thermal properties of Mo*_x_*W_(1‐_ *_x_*_)_Se_2_^[^ ^19^ ^]^ and MoS_2(1‐_ *_x_*_)_ Se_2_ *_x_*,^[^ ^20^, ^21^ ^]^ or the transformation between the Mott‐insulating and metallic phase in 1T‐TaS_2‐_ *_x_*Se*_x_*.^[^ ^22^ ^]^ Optimizing doping parameters, including doping concentrations, nature of dopants (e.g., metallic or nonmetallic, transition metals, chalcogen, halogen or pnictogen^[^ ^23^ ^]^), and doping types (e.g., substitutional doping, interstitial, or physical adsorption) is essential to merit the positive effects on the performance efficiency, durability, and sensitivity of the 2D materials.^[^ ^24^ ^]^ However, the options of substituents need to be carefully evaluated because they could impair the properties of 2D TMDs for practical applications by creating deep trap states or destabilizing the structures. Therefore, the relevant scientific questions come down to: 1) What elements can be incorporated into a particular 2D TMD? 2) What is energetically favorable configuration for the dopant in the TMD lattice? 3) How would the dopant modify the fundamental properties and functionality in the TMDs? These questions stimulate theoretical investigations into the doping mechanisms, energetics, and electronic structures by first principle calculations.

Many informative literature reviews focus on new applications and novel heterogeneities from synthetic 2D TMD induced by doping and alloying.^[^ ^25^, ^26^, ^27^, ^28^ ^]^ So far, the focus of most reviews leans toward the materials produced by solid‐source chemical vapor deposition (CVD_SS_) that uses powder precursors and salt promoters vaporized at high temperature. This method is versatile for a whole library of 2D TMDs and various metastable phases,^[^ ^29^ ^]^ and can provide large‐size, single‐crystalline 2D domains on SiO_2_/Si or sapphire rapidly within an hour. While CVD methods are arguably the most popular method for prototyping novel 2D materials for research at a laboratory level, as typically employed these methods lack proper controllability in terms of reaction pathways, spatial uniformity, doping concentrations, and therefore reproducibility required for practical and large‐scale manufacturing. To accelerate their technological readiness, it is imperative to extend the efforts of this thrust to more controllable and industrially acceptable thin‐film methods.^[^ ^30^ ^]^ In this progress report, we will begin with the theoretical overview of the formation energy, band structures, and energetics of doped and alloyed TMDs that can guide experimentalists to choose desirable dopants for particular TMDs. We will put special emphasis on non‐equilibrium thin‐film techniques that can provide scalable, large area synthesis and also good degrees of controllability for the compositional engineering of TMDs, followed by reviewing the selected work of doped and alloyed TMDs achieved by both bottom–up and top–down methods. At the end, conclusion and perspective will be drawn and highlight some challenges that one may encounter while using these techniques on controlling the atomic compositions of 2D TMDs.

## 2 Theoretical Guidance for Doping and Alloying TMDs

A throughput computational analysis is essential to guide experimentalists toward which elements on the periodic table properly dope TMDs. Density functional theory (DFT) calculations predict the formation energies, optimized atomic geometry, and electrical properties of each elemental dopant in TMDs (Figure 1a).^[^ ^31^, ^32^ ^]^ The general calculation of the formation energies (*E*_f_) is expressed as *E*_f_ = *E*_(TMD + Dopant)_ − *E*_(TMD)_ − *μ*_(Dopant)_, where E_(TMD + Dopant)_ and E_(TMD)_ are the total energies of the supercell with the dopant and pristine TMD monolayer, and *μ*_(Dopant)_ is the chemical potential of the dopant. The equation can be further modified for *E*_f_ under metal‐rich or chalcogen‐rich conditions. Onofrio et al.^[^ ^32^ ^]^ examine each dopant in either metal‐rich or chalcogen‐rich chemical potential conditions, and highlight that elements with formation energy (*E*_f_) < 1 eV or with negative *E*_f_ are promising for the substitutional doping of Group VI TMD monolayers for electronic applications (Figure 1a–c). Elements adjacent to Group VI transition metal (TM) and Group XVI chalcogen constituents of 1H‐TMDs are easier to incorporate into the lattice due to similar atomic size and electronegativity. Furthermore, incorporating elements from Group V and VII for the M sites, and Group XV and XVII for the X sites, may modify band structures and Fermi level energy without introducing deep charged states (Figure 1c). Conversely, the elements that are predicted to be energetically unfavorable for substitutional doping may still be introduced into TMDs by non‐equilibrium approaches. Most Group I and II elements have a high *E*_f_ either at M or X sites and therefore are not energetically favorable for substitutional doping. The alternative approaches can be surface functionalization (e.g., degenerately doped WSe_2_ with K)^[^ ^33^ ^]^ or interlayer intercalations (e.g., Li, Na, and Mg).^[^ ^34^ ^]^ Similarly, some metal elements with high *E*_f_ in Group IX, X, and XI, such as Ir, Pd, Ni, and Au, can be thermally deposited on TMD devices as electrical contacts, facilitating either p‐type or n‐type charge transport.^[^ ^35^ ^]^ If there are existing chalcogen vacancies,^[^ ^36^, ^37^ ^]^ many TM elements (except those in Group X–XII^[^ ^37^ ^]^) could fill in the vacancies to substitute the X sites as an energetically preferable choice.

Karthikeyan et al. theoretically demonstrated how TM adatoms deposited on the surface of MoS_2_, MoSe_2_, and MoTe_2_ were incorporated into the lattices, following the most energetically favorable pathway.^[^ ^37^ ^]^ The possible final configurations, including adsorption on top of the Mo atoms, chalcogen site substitution, and stabilization in the interstitials, were evaluated (Figure 1d). While substitutional doping is energetically favorable for many impurities on MoS_2_ and MoSe_2_, interstitial doping is easier than substitutional doping to achieve for 3d TM atoms (from Sc to Zn) on MoTe_2_ due to its relatively sizeable primitive cell that provides more free space to the interstitial atoms than MoS_2_ and MoSe_2_ (1st row in Figure 1d). It is worthy of reviewing the theory behind the interstitial doping of MoTe_2_ briefly because some of TM elements at the interstitial sites could raise ferromagnetisms in monolayers. The first few 4d and 5d TM elements (the 2nd and 3rd rows in Figure 1d) could substitute the Mo atom sites as the most stable configuration and displace the Mo atoms to the nearest voids. The hybridization of the d‐orbitals of these doped TM atoms and the d‐orbitals of their surrounding Mo atoms results in bonding orbitals and nonbonded d‐orbitals. After bonding orbitals are filled, electrons start to occupy the nonbonded orbitals. As a result, the magnetic moment increases from V to Mn and then decreases due to the electron pairing. Therefore, the interstitial Mn, Tc, and Re in MoTe_2_ with three unpaired electrons in the unbonded d‐orbitals provide the largest magnetic moment. This post‐synthesis doping strategy using dispersed TM atoms provides a unique capability to create metastable configurations and localized magnetic moments that cannot be attained by high‐temperature synthesis or energetic ion implantation. A controllable deposition from the molecular beam epitaxy (MBE) can deposit TM atoms in vacuum onto MoTe_2_ to experimentally achieve doping at interstitial sites.^[^ ^38^ ^]^

**Figure 2:**
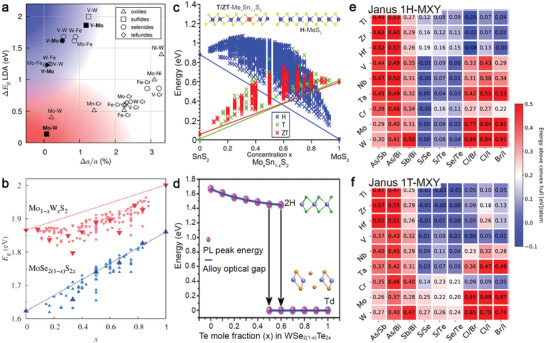
Theoretical prediction for 2D TMD alloys. a) Lattice constant matching for transition metal pairs of H‐phase TMDs based on local density approximation lattice constants. The upper left corner of the plot is populated with metal–semiconductor alloys, such as V–Mo and V–W. The lower‐left corner contains semiconductor–semiconductor alloys of molybdenum and tungsten dichalcogenides. The logos in the legend are denoted for the type of chalcogen constituents in the compounds. Reproduced with permission.^[^ ^40^ ^]^ Copyright 2014, The Royal Society of Chemistry. b) A plot of theoretically predicted band gaps in Mo_1‐_ *_x_*W*_x_*S_2_ and MoSe_2(1‐_ *_x_*_)_S_2_ *_x_* alloys versus concentration *x*. The band gaps in the thermodynamic ground states at different *x* are shown with larger symbols. Reproduced with permission.^[^ ^40^ ^]^ Copyright 2014, The Royal Society of Chemistry. c) A SnS_2_−MoS_2_ phase diagram of the predicted energies from the cluster expansion methods for H‐, T‐ and ZT (*T*_d_)‐phase Sn_1‐_ *_x_*Mo*_x_*S_2_ alloy shows T‐phase in MoS_2_ could be stable when *x* = 0.2. Reproduced with permission.^[^ ^43^ ^]^ Copyright 2016, American Chemical Society. d) The composition‐dependent bandgaps and crystal phase of WSe_2(1‐_ *_x_*_)_Te_2_ *_x_* crystals show a transition from 1H to *T*_d_ phase at *x* = 0.5–0.6. Reproduced with permission.^[^ ^44^ ^]^ Copyright 2017, Wiley‐VCH. e,f) Thermodynamic stability of the 1H‐phase and 1T‐phase for different MXY Janus monolayers. The colors denote the energy above the convex hull in ev per atom. Reprinted with permission.^[^ ^37^ ^]^ Copyright 2019, American Chemical Society.

The doping concentration is typically at the percentage level when the dopant starts to impact the electronic properties of TMD devices due to confinement effects and enhanced dielectric screening of the 2D layer.^[^ ^27^, ^39^ ^]^ Besides, 2D TMDs can incorporate large amounts of foreign elements, becoming alloys. The path finding tasks via theoretical computation can help identify thermodynamically stable 2D alloys in various configurations. To narrow the range for finding practical TMD binary alloys, Kutana et al. suggest a few selection rules:^[^ ^40^ ^]^ 1) the lattice mismatch of the lattice constants between the two chosen TMDs should be smaller than 3.4%; 2) the difference between their metal‐chalcogen bonds should be smaller than 0.1 Å; and 3) one chosen TMD component needs to have non‐zero bandgap.^[^ ^40^ ^]^ **Figure** **2a** ased on the selection rules, indicates that metal–semiconductor TMD alloys (e.g., V–Mo dichalcogenides) could provide a wide‐range tunable bandgap and have a small lattice mismatch that makes them more likely obtainable. The cluster expansion (CE) method provides a full microscopic description of atomic configurations in a crystal, and is used to calculate the formation enthalpy of a ternary TMD alloy across a range of alloying ratios.^[^ ^40^, ^41^, ^42^ ^]^ Group VI alloys, such as Mo_1‐_ *_x_*W*_x_*S_2_^[^ ^40^ ^]^ and MoS_2(1‐_ *_x_*_)_Se_2_ *_x_*,^[^ ^42^ ^]^ are arguably the most favorable semiconducting alloys because of their linearly tunable direct bandgap (Figure 2b) and good thermodynamic stability at any alloying ratio.^[^ ^42^ ^]^ From an experimental perspective, Mo_1‐_ *_x_*W*_x_*S_2_ is readily grown using different precursor ratios or via post‐growth sulfurization/selenization to make MoS_2(1‐_ *_x_*_)_Se_2_ *_x_*. Forming TMD alloys can also stabilize the metastable crystal phase in pristine TMDs. For example, a 1T‐structure is challenging to make in MoS_2,_ whose ground state is in the 1H‐phase. A combination of DFT and CE calculations performed on Mo*_X_*Sn_1‐_ *_x_*S_2_^[^ ^43^ ^]^ suggests that alloying 20% Mo with Sn could lower the *E*_f_ for 1T structure by 50%. Similarly,1H‐WSe_2(1‐_ *_x_*_)_Te_2_ *_x_* is less stable with increasing Te incorporation.^[^ ^41^ ^]^ Experimentally, Te‐doped WSe_2_ is found to transform into the distorted octahedral structure (*T*_d_) when Te exceeds Se to overcome the instability, while exhibiting metallic behavior (Figure 2d).^[^ ^44^ ^]^

Janus 2D TMDs consist of different chalcogens occupying the top and bottom of a monolayer that result in the structural symmetry breaking. The lack of mirror symmetry induces a permanent dipole moment that could enhance their functionalities, such as piezo‐response, catalytic behavior, and electron–hole separation. They could also create interesting effects when stacked with other 2D materials to form heterostructures, such as tuning interlayer excitonic dynamics, Schottky barrier, and band alignment at the interfaces. While Janus MoSSe and WSSe monolayers and their properties have been explored, there are plenty of unknown yet interesting Janus TMDs waiting to be explored. Riis‐Jensen et al., systemically investigated MXY Janus structures in both H‐phase and T‐phase and accessed the thermodynamic stability of 216 monolayers from their calculated energy above convex hull (*E*_hull_) per atom (Figure 2e,f).^[^ ^45^ ^]^ If the *E*_hull_ per atom for one MXY Janus monolayer is too high, it is unstable and will decompose into other phases upon successful synthesis. Overall, the stable structures worthy of experimental exploration in the H‐phase are M with one of Group IV–VI elements, and both X and Y with one chalcogen element. In T‐phase, they are M with one of Group IV–V elements, and both X and Y with one chalcogen element. Therefore, this information regarding Janus monolayers' stability with different atoms and between two crystal phases can help experimentalists rule out the Janus monolayers too challenging to synthesize and preserve.

## 3 Thin‐Film Techniques for the Compositional Engineering of 2D TMDs

The initial research on compositionally engineered 2D TMD used exfoliated monolayers and few‐layers frequently from synthetic bulk crystals to ensure the highest material quality.^[^ ^20^, ^46^ ^]^ Bulk crystal growth techniques include vapor transport, flux growth, and directional solidification like the Bridgeman–Stockbarger method.^[^ ^47^ ^]^ Equilibrium phase diagrams used to understand the phases under different chemical conditions is a starting point for crystal growth.^[^ ^47^ ^]^ These equilibrium methods grow crystals in a vacuum‐sealed quartz ampoule. Inside the ampoule, multiple elements of the targeted crystals melt and regrow into a crystal at high temperature over days and weeks during which temperature and pressure are constant to let melted elements find the most energetically favorable sites to attach and enlarge the growing crystals uninterruptedly. Although bulk crystal growth can provide the highest quality crystals for fundamental research, it is not suitable to exploit heterogeneity in 2D crystals rapidly or grow large area 2D samples for electronic, catalysis, and energy applications. To synthesize 2D TMDs more scalably and flexibly, thin‐film techniques, including chemical vapor deposition (CVD), physical vapor deposition (PVD), atomic layer deposition (ALD), molecular beam epitaxy (MBE), and pulsed laser deposition (PLD) are adequate alternatives.^[^ ^30^ ^]^ These systems involve the flux of energy and matter and chemical reactions^[^ ^48^, ^49^ ^]^ and are labeled as non‐equilibrium techniques. For example, CVD uses volatile precursors that react and produce clusters and particles on the surface that will evolve into a crystal;^[^ ^50^, ^51^ ^]^ PLD uses laser‐ablation plasmas that deliver energetic species with kinetic energies (KE) of 1–100 eV per atom.^[^ ^52^ ^]^ The processing parameters can be tuned to control metastable phases, grain boundaries, and defects in 2D crystals that help reduce the energy barriers of substitutional doping for a broader range of dopants.

**Figure 3:**
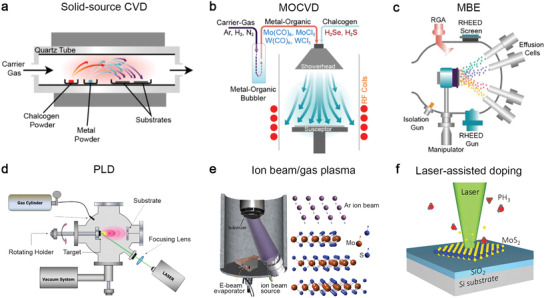
Thin‐film techniques for achieving doped and alloyed 2D TMDs. a) Solid source chemical vapor deposition (CVD_SS_) evaporates multiple powder precursors at high temperature and deposits 2D crystals on substrates placed downstream. Reproduced with permission.^[^ ^30^ ^]^ Copyright 2019, IOP Publishing. b) Metal‐organic chemical vapor deposition (MOCVD) uses volatile precursors delivered into the reactor by carrier gas flowing through bubbler manifolds where MO precursors are stored. Reproduced with permission.^[^ ^30^ ^]^ Copyright 2019, IOP Publishing. c) Molecular beam epitaxy delivers multiple elemental sources at ultra‐high vacuum using the Knudsen effusion cells. RHEED and RGA are standard techniques in MBE for monitoring thickness and environmental impurity. Reproduced with permission.^[^ ^30^ ^]^ Copyright 2019, IOP Publishing. d) Pulsed laser deposition uses plasma plumes created by laser ablation of a target using an excimer laser (e.g., 248 nm, 25 ns pulse width) to deliver stochiometric precursors to the substrate in elevated temperatures. Reproduced under the terms of CC‐BY license.^[^ ^53^ ^]^ Copyright 2020, The Authors, published by MDPI. e) Top–down, ion beam‐ or plasma‐assisted processes convert as‐grown pristine TMD into doped/alloyed samples by generating defects and supplying dopants to fill in the defects. Reproduced with permission.^[^ ^89^ ^]^ Copyright 2019, American Chemical Society. f) Top–down laser‐assisted chemical vapor doping of as‐grown TMDs at room temperature. The opto‐thermal energies provided by a laser can dissociate the gaseous molecules comprised of dopants and create defects on TMDs. Reproduced with permission.^[^ ^13^ ^]^ Copyright 2016, Wiley‐VCH.

Here, we briefly describe the characteristics of each thin‐film technique (**Figure** **3**).^[^ ^13^, ^30^, ^53^, ^54^ ^]^ Solid‐source CVD (CVD_SS_)^[^ ^10^, ^55^ ^]^ uses powder comprised of transition metals (TM) and chalcogens as the raw materials to fabricate 2D crystals above 550 °C.^[^ ^29^ ^]^ For example, MoO_3_, WO_3_, Re_2_O_5_ powders supply TM, and S, Se, and Te powders supply chalcogens (Figure 3a). CVD_ss_ usually does not require a lattice‐matched substrate to grow 2D crystals and is capable of growing hundreds of micrometers to millimeter‐sized domains on silica. Due to the simplicity of setting up CVD_ss_ at the lab and ability to provide large single‐crystalline domains, compared to other deposition techniques, it is frequently used to exploit all variants in the library of 2D TMDs.^[^ ^29^ ^]^ Metal‐organic chemical vapor deposition (MOCVD) and ALD use volatile precursors and gaseous molecules to grow and dope 2D TMDs (Figure 3b). Mo(CO)_6_, W(CO)_6_, or NbCl_5_ are volatile and can be delivered into the system by flowing controllable carrier gas by regulating the temperature and pressure of the bubblers where they are stored. H_2_S, H_2_Se, diethyl sulfide (C_4_H_10_S), and diethyl telluride (C_2_H_6_Te) are used to supply chalcogens for growing 2D TMDs. To reduce impurities and carbon contamination, it uses large H_2_ concentrations in the system during deposition. Typically, MOCVD requires lattice‐matched substrates, such as sapphire and GaN, for growing epitaxially aligned 2D TMDs.^[^ ^51^, ^56^ ^]^ It can grow good TMDs on vdW substrates, such as graphene, boron nitride, and TMDs by vdW epitaxy at high temperature (≥ 800 °C).^[^ ^57^ ^]^

The temperature and pressure of CVD_SS_ are typically limited by the ambient vapor pressures and melting temperatures of transition metal and transition metal oxide precursors. For example, the melting temperature and ambient vapor pressure of MoO_3_ is 800 °C and 10^−6^ Torr, respectively. Therefore, synthesizing MoS_2_ or MoSe_2_ via CVDss typically requires 600–900 °C of the growth temperature. The common precursors of the transition metals (Mo(CO)_6_ and W(CO)_6_, WCl_6_) and the chalcogens (H_2_S, H_2_Se, and (CH_3_)_2_Se) for growing TMDs have high ambient vapor pressures and can dissociate below 400 °C.^[^ ^58^, ^59^, ^60^ ^]^ The substrate temperature needs to be high enough (i.e., >700 °C)^[^ ^56^ ^]^ to overcome the kinetic limitations, including the balance between the adsorption and desorption and surface diffusion of adatoms to obtain electronic grade materials.^[^ ^61^, ^62^ ^]^ On the other hand, it can also grow 2D TMDs in polycrystalline forms at 450 °C and is suitable for the back‐end‐of‐line (BEOL) integration in Si technology thanks to the high reactivity of metal‐organic precursors.^[^ ^63^ ^]^

MBE is a promising technique for growing TMDs due to the potential of enhanced crystal quality enabled by a combination of high‐purity elemental sources and an ultra‐high vacuum (UHV) growth environment (Figure 3c). It uses effusion cells for supplying chalcogens and e‐beam evaporators for supplying TM. Its deposition temperature for TMDs is between 300 and 600 °C; The background pressure is around 10^−9^ mbar during growth. Although MBE typically produces small grain sizes due to limited kinetic processes and high nucleation density, it can grow a full range of 2D alloys with fine controllability, such as WTe_2‐_ *_x_*Se*_x_* and V_1‐_ *_x_*Mo*_x_*Se_2_,^[^ ^64^, ^65^ ^]^ for the study of stability and phase transformation. PLD (Figure 3d) is a versatile technique to explore the deposition and implantation of advanced thin films by the laser ablation of a high‐density solid target using a pulsed excimer laser (25 ns, 248 nm).^[^ ^52^, ^53^ ^]^ The kinetic energy (KE) of species in laser‐ablation plasmas used for PLD can be as high as 100 eV in a vacuum (e.g., amorphous carbon from the ablation of graphite),^[^ ^52^ ^]^ allowing the exploration of defects and metastable phases in TMDs.^[^ ^66^, ^67^ ^]^ To reduce the KE of the species in plasmas, the gaseous molecules such as Ar and O_2_ are introduced into the system from a gas cylinder to confine and slow the species. For growing, doped/alloyed TMD thin films, the ablation targets in PLD are made of targeted compounds with pre‐determined atomic ratios.

The chalcogen/transition metal (C/TM) ratio during the synthesis can control domain morphology and stoichiometry. For example, a chalcogen‐rich (metal‐rich) growth environment is thermodynamically favorable for triangular (hexagonal) TMD domains. Experimentally, Yue et al. found that a chalcogen‐poor environment in MBE could result in island growth or metal cluster growth.^[^ ^68^ ^]^ The first principle calculations also suggest that the formation energy for various defects in MoS_2_ monolayer is dependent on the chemical potential of the chalcogen, which can be controlled by the C/TM ratio in a growth environment.^[^ ^69^ ^]^ Additionally, the growth pressure of CVD can impact domain size, domain density, and the number of carbon atoms that incorporate into grown layers.^[^ ^70^, ^71^, ^72^, ^73^, ^74^ ^]^ The change in growth pressure also changes the sticking efficiency of adatoms on the surface and the impurity concentration (i.e., water vapors^[^ ^73^ ^]^ or carbons dissociated from metal‐organic precursors^[^ ^71^, ^75^ ^]^). Eichfeld et al. studied the pressure effect for MOCVD grown WSe_2_ on graphene at 750 °C in a vertical cold‐wall reactor and found WSe_2_ domain density reduces, and domain size increases when the pressure increases from 500 to 700 Torr.^[^ ^70^ ^]^ It is possible that the adatoms cannot stick on the surface long enough to diffuse and incorporate into existing domains to enlarge them at lower pressures.

Most of these bottom–up methods, including PLD, MOCVD, and MBE, can combine in situ characterizations to monitor film thickness, precursor chemistry, impurities, and growth kinetics. This data logging could help understand growth conditions on‐the‐fly and allows us to revise growth parameters during growth. For example, laser reflectivity (LR),^[^ ^66^ ^]^ spectroscopic ellipsometry (SE),^[^ ^76^ ^]^ and reflection high‐energy electron diffraction (RHEED)^[^ ^64^, ^77^ ^]^ can monitor layer‐by‐layer growth of TMDs, providing growth rates, optical properties, and crystal phases of a growing crystal. While RHEED requires a UHV environment to operate, LR and SE are mounted outside of systems with viewports using optical beams and photodetectors. Plasma measurements, including ion probes and intensified CCD cameras for time‐resolved imaging spectroscopy, can monitor the cluster sizes, excitation states, and kinetic energies of the species in laser‐ablation plasmas in PLD.^[^ ^78^ ^]^

Ion implantation and defect‐assisted doping rely on the exchange of mass, energy, and momentum with the constituents to generate vacancies for dopants to fill in (Figure 3e). Ion implantation can achieve these tasks within a single step using energetic ions. The ion's species, energy, flux, and angle of incidence can be customized. An ion source for implantation generates ions with energies in the range of 10–500 keV, sufficient to create a penetration depth between 10 nm and 1 µm upon implantation. It requires post‐implantation annealing to heal defects and secure the implanted dopants in the lattice. However, it is impractical to implant 2D TMDs with standard high‐energy ion implantation because it could damage materials or penetrate them directly. In response to this problem, several works demonstrated low‐energy implantation to reduce damages during doping 2D crystals.^[^ ^12^, ^67^, ^79^ ^]^ Reactive plasmas of N_2_, H_2_, and O_2_ have been used to achieve single‐step substitutional doping. On the other hand, defect‐assisted doping is a two‐step process that creates vacancies in 2D crystals with ion beam bombardment or gaseous plasmas and fills in the vacancies with doping sources introduced afterward. This approach has been used for electrical contact in devices^[^ ^54^ ^]^ or selectively converts a 2D TMD to 2D Janus structures.^[^ ^80^, ^81^ ^]^

Direct laser writing is of great interest due to its versatility for spatially growing and engineering the chemical compositions of 2D crystals. It has been used with chemical vapors as a one‐step approach to dope TMDs at room temperature in an environmental chamber (Figure 3f).^[^ ^13^, ^82^ ^]^ It can optothermally evaporate the constituents of TMDs and generate vacancies. It can simultaneously dissociate volatile molecules introduced into the chamber to provide dopants to fill in the vacancies. Laser‐assisted doping can conveniently combine laser spectroscopy that monitors dynamic spectra evolution in terms of defect generation, layer thinning,^[^ ^83^ ^]^ and compositional evolution,^[^ ^82^ ^]^ to observe the transformation directly. Besides, direct laser writing can crystallize as‐deposited amorphous precursors mixed of dopants and constituents with predetermined molar ratios into doped/alloyed TMDs.^[^ ^84^ ^]^

This report consolidated several selected recent works concerning the substitutional doping and alloying of 2D TMDs in each thin‐film technique. In particular, the substitutional doping techniques have advanced significantly in the past few years and are gradually phasing out the unreliable surface functionalization methods for industrial application.^[^ ^85^ ^]^ Compared to equilibrium bulk crystal growth, they can incorporate more types of dopants into the crystals or achieve metastable structures more prone to dopants than stable structures due to their non‐equilibrium nature. Each technique provides a unique capability to meet our desires for increasing heterogeneities or modulating physical and chemical properties in 2D TMD crystals. In each technical section, the corresponding characterization tools and the properties are discussed to understand recently doped and alloyed samples. Although this work does not cover novel heterogeneities and electrical properties in doped and alloyed TMDs in detail, there are recently published review articles^[^ ^27^, ^28^, ^86^, ^87^, ^88^ ^]^ specifically focusing on those aspects of doped 2D crystals and 2D alloys on demand to the readers.

## 4 Bottom–Up Approaches

### 4.1 Solid‐Source Chemical Vapor Deposition

**Figure 4:**
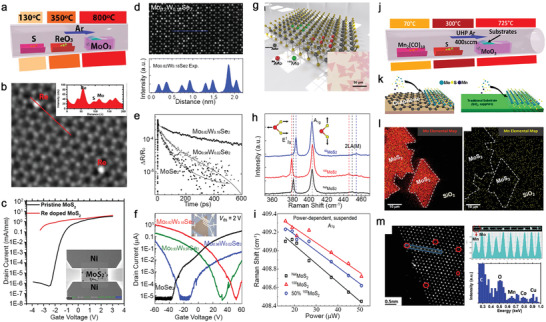
Solid‐source CVD for compositionally engineering 2D TMDs. a) A schematic of the CVDss for Re‐doped MoS_2_ monolayer describes precursor positions and the temperatures required for each precursor powder. Reproduced with permission.^[^ ^10^ ^]^ Copyrgiht 2018, Wiley‐VCH. b) An annular dark‐field STEM (ADF‐STEM) image shows Re atoms identified by the Z‐contrast intensity substituted Mo sites shown in the inset. Reproduce with permission.^[^ ^10^ ^]^ Copyright 2018, Wiley‐VCH. c) Comparison of the transfer characteristics (at *V*_ds_ = 0.5 V) based on pristine and Re‐doped MoS_2_ indicates the negative shift of the threshold voltage and lack of gate tunability on Re‐doped (1%) MoS_2_ FET. Reproduced with permission.^[^ ^10^ ^]^ Copyright 2018, Wiley‐VCH. d) ADF‐STEM image of Mo_0.82_W_0.18_Se_2_ indicates that the brightest spots are W substituting at Mo sites. The experimental Z‐contrast intensity profiles along the blue line in the image show the Z‐contrast intensity differences between W, Mo, and Se_2_. Reproduced with permission.^[^ ^91^ ^]^ Copyright 2016, Wiley‐VCH. e) Differential reflection signal measured from pristine and alloy monolayers with a 620 nm pump and an 800 nm probe pulse indicate the extended measured lifetime of monolayers with increased W concentrations. Reproduced with permission.^[^ ^92^ ^]^ Copyright 2017, Wiley‐VCH. f) Transfer characteristics (at *V*_ds_ = 2 V) of FET based on pristine and alloy monolayers with different W concentrations. Reproduced with permission.^[^ ^91^ ^]^ Copyright 2016, Wiley‐VCH. g) Schematic of MoS_2_ monolayer composed of different Mo isotopes (Mo^92^ and Mo^100^). Reproduced with permission.^[^ ^93^ ^]^ Copyright 2019, American Chemical Society. h) Raman spectra of ^Nat^MoS_2_, ^92^MoS_2_, and ^100^MoS_2_ monolayers acquired using a 532 nm laser show isotope effects dictate the in‐plane *E* ^1^_2g_ peak position. Reproduced with permission.^[^ ^93^ ^]^ Copyright 2019, American Chemical Society. i) Laser excitation‐power dependent frequency of *A*_1g_ phonon mode of suspended ^Nat^MoS_2_, ^92^MoS_2_, and 50% ^100^MoS_2_ monolayers measured at room temperature. The thermal conductivity of MoS_2_ monolayers increases with increasing isotope concentration. Reproduced with permission.^[^ ^93^ ^]^ Copyright 2019, American Chemical Society. j) Synthesis of Mn‐doped MoS_2_ uses Mn_2_(CO)_10_ and S located upstream of the hot zone, and MoO_3_ and growth substrate located in the hot zone. Reproduced with permission.^[^ ^16^ ^]^ Copyright 2015, American Chemical Society. (*k*) The substrates for comparison include graphene and insulating substrates (sapphire and SiO_2_). Reproduced with permission.^[^ ^16^ ^]^ Copyright 2015, American Chemical Society. l) The elemental analysis of the doped MoS_2_ by TOF‐SIMS provides evidence that the concentration of Mn (yellow dots) on the substrate is equivalent or higher to that found in the areas with MoS_2_, indicating that Mn may be bound to the substrate surface, instead of being incorporated into the MoS_2_ lattice. Reproduced with permission.^[^ ^16^Copyright 2015, American Chemical Society.m) STEM experiments show that the Mn is incorporated at the MoS_2_ domain boundary and at the Mo sites. Each Mn atom is identified from the Z‐contrast intensity spectra of the selected area, where the Mn exhibits the intensity half than that of Mo. Energy‐dispersive X‐ray spectroscopy of the Mn region exhibits a weak Mn signal, which is expected due to the detection limit of EDX. Co and Cu signals come from the TEM grid. Reproduced with permission.^[^ ^16^ ^]^ Copyright 2015, American Chemical Society.

This method evaporates raw materials in powder form at high temperature and grows TMD crystals on substrates. It is the most popular choice in this research field because it is simple to set up a horizontal tube reactor and does not require sophisticated pressure and gas flow control. The charge carrier type and concentration of the TMD monolayers that combine Group IV–VI elements can be modulated between n‐type, isoelectronic, and p‐type doping. Therefore, they are of great interest to transistors, low‐power electronics, and photonic devices. Zhang et al. demonstrated Re‐doping of synthetic MoS_2_ by adding ReO_3_ into the growth that uses MoO_3_ and S powders for MoS_2_ (Figure **4a**).^[^ ^10^ ^]^ ReO_3_ is ideal as the precursor for Re because it can be vaporized at 380 °C, while MoO_3_ requires high temperature. r‐phase sapphire was chosen as the substrate for a deposition because it reduces film–substrate interaction, thereby improving Re concentration in the film. X‐ray photoemission spectroscopy (XPS) quantitatively confirmed the 1 at% Re concentration in Re‐MoS_2_ monolayer. Annular dark field‐scanning transmission electron microscopy (ADF‐STEM) was used to study the Re incorporation at the atomic scale and found them substituted at Mo sites according to the brightest Z‐contrast intensity (Figure 4b and inset). The field‐effect transistors (FET) fabricated on both Re‐MoS_2_ and pristine MoS_2_ were compared (Figure 4c), which indicate the doping level of 1% can push the MoS_2_ to be degenerately n‐doped, evident by a large negative threshold voltage shift (>2 V) and a lack of gate control over a voltage range >6 V. Their claim was further supported by the DFT calculations and conductive atomic force microscopy (C‐AFM) measurement.^[^ ^10^ ^]^

Mo and W can be mixed to create isoelectronically doped TMD semiconductors. For example, Li et al. synthesized W‐doped MoSe_2_ (Mo*_x_*W_1‐_ *_x_*Se_2_) using mixed WO_3_/MoO_3_ powder as precursors.^[^ ^90^, ^91^, ^92^ ^]^ The mixing ratio between W and Mo (0 < *x* < 0.18) can be tuned on grown layers by controlling the precursor ratios of WO_3_/MoO_3_. Figure 4d shows an ADF‐STEM image of a homogenous Mo_0.82_W_0.18_Se_2_ alloy where W stands out because of its highest Z‐contrast intensity, among other atoms (see Z‐contrast intensity profile in Figure 4d). One great advantage of W‐MoSe_2_ is that the deep trap states within the bandgap of MoSe_2_ associated with Se vacancies can be alleviated by adding low W concentrations whose W—Se bond can increase the formation energy of Se vacancies. This was further confirmed by an ultrafast exciton dynamics measurement performed on MoSe_2_, Mo_0.98_W_0.02_Se_2_, and Mo_0.82_W_0.18_Se_2_ monolayers (Figure 4e). Their exciton dynamics show that the lifetime decays in terms of the intra‐band energy relaxation process by phonon scattering (30, 50, and 65 ps) and exciton lifetime (116, 244, and 389 ps) increase with higher W concentrations, implying the reduction in defect‐induced nonradiative recombination centers originated from Se vacancies. Unlike Re and other group‐VII that would provide extra electrons to the MoSe_2_, isoelectronic dopants like W, in this case, utilize electronegativity to modulate the carrier density for non‐degenerate doping. The FETs of Mo*_x_*W_1‐_ *_x_*Se_2_ monolayers (Figure 4f) show a positive shift in the p‐type threshold voltage from −48, −18, to −12 V as the W concentrations increased from 2%, 7% to 18%. This indicates the increased W doping, not only suppresses n‐type transport but also enhances the p‐type transport.

Recently, the isotope effect has emerged as a tuning knob for the physical properties of 2D TMDs while preserving their material chemistry.^[^ ^93^, ^94^ ^]^ Therefore, it is worth considering isotope effects as part of doping and alloy strategies for TMD crystals. Isotopes of an atom have the same electron number but vary in neutron number and atomic mass. In a solid‐state crystal, the resulting variation in the nuclear mass will influence the phonon properties that govern the thermal, electronic, and vibrational behaviors.^[^ ^95^ ^]^ Like naturally abundant 2D TMDs, isotopically pure or isotopically alloyed TMD monolayers can also be prepared by CVD_SS_. Li et al. used isotopically enriched MoO_3_ powders that contain ^92^Mo or ^100^Mo with 98% enrichment to grow ^92^MoS_2_, ^100^MoS_2_, and 50% ^100^MoS_2_ with half of Mo belong to naturally abundant ones (Figure 4g).^[^ ^93^ ^]^ The use of isotopically enriched MoO_3_ does not alter the size of grown MoS_2_ monolayers (Figure 4g inset) and leads to their quality improvement according to the photoluminescence and exciton lifetime measurements. The Raman spectra of ^92^MoS_2_ and ^100^MoS_2_ show a red‐shift of their in‐plane vibrational mode (*E* ^1^_2g_), in which the Mo vibration is involved, from 385.7 to 380.7 cm^−1^ as the mass of Mo increases from 92 to 100 (Figure 4h). Besides, the in‐plane thermal conductivity of suspended naturally abundant MoS_2_ (^Nat^MoS_2_), 50% ^100^MoS_2_, and ^100^MoS_2_ monolayers were measured using an optothermal Raman technique to understand the impact of isotope purity on their thermal transport. Figure 4i shows laser power‐dependent, red‐shifted linear curves of all *A*_1g_ modes whose slopes are used to calculate the power coefficient (*χ*_P_). The same methods obtained their temperature coefficient (*χ*_T_) by computing with the increased temperature. With *χ*_P_ and *χ*_T_, their thermal resistance (*R*_m_ = *χ*_P_/ *χ*_T_) would be known and used to extract their in‐plane thermal conductivity (*κ*). The *κ* of ^Nat^MoS_2_, 50% ^100^MoS_2_, and ^100^MoS_2_ are 41, 53, and 62 W mK^−1^, respectively. The latter two show a near 50% and 30% enhancement compared with the ^Nat^MoS_2_, indicating the isotopically pure 2D materials can reduce phonon scattering, thereby improving the thermal properties of monolayers.^[^ ^93^ ^]^

Doping magnetic elements such as manganese (Mn) and iron (Fe) can add new functionalities such as magnetism and spin into non‐magnetic TMDs through CVD_SS_.^[^ ^14^, ^16^ ^]^ Although DFT calculations already indicate that the *E*_f_ for doping both Mn and Fe in H‐phase Group VI TMDs is small and negative, indicating their doping is achievable, the choice of substrates could decide their successful incorporation. Zhang et al. synthesized Mn‐MoS_2_ with an Mn concentration < 2% using Mn_2_(CO)_10_ to supply Mn in the CVD_SS_ for MoS_2_ monolayers grown on graphene and SiO_2_/Si (Figure 4j). Interestingly, they found that Mn can successfully incorporate into MoS_2_ on graphene but could not do so on SiO_2_ or sapphire under the same growth conditions (Figure 4k). To verify if any Mn was incorporated, time‐of‐flight second ions mass spectroscopy (TOF‐SIMS) that can detect Mn on Si with a sensitivity of 10^9^ cm^−2^ was carried out on presumably Mn‐doped MoS_2_ on SiO_2_ (Figure 4l). The analysis pointed out Mn's relative similarity in the MoS_2_ domains versus off‐MoS_2_ regions, which indicates that Mn is bonding to Si instead of being incorporated into the MoS_2_. It is possible that during the growth, reactive Mn could passivate the dangling bonds on the silica surface, leading to Mn—Si bonding that is more energetically preferable than Mn incorporation into MoS_2_. Conversely, it was found that Mn‐MoS_2_ grown on suspended graphene has Mn that either substituted Mo or attached to the domain edges, as confirmed by ADF‐STEM and electron diffraction X‐ray (EDX) measurement (Figure 4l,m). Although this work did not prove the magnetic properties in Mn‐doped MoS_2_ on graphene due to only a small amount of Mn incorporation, it provides evidence that the surface chemistry and precursor reactivity could pose a significant experimental challenge for doping TMDs with the elements that may not be energetically preferable for TMDs like Mn.^[^ ^16^ ^]^

### 4.2 Metal‐Organic Chemical Vapor Deposition

**Figure 5:**
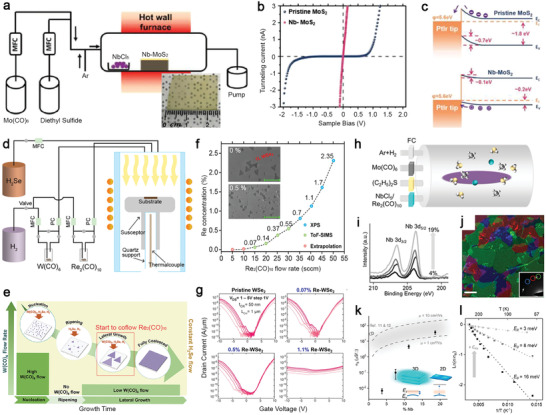
Examples of doped 2D TMDs grown via the MOCVD process. a) Schematic of the hot‐wall hybrid MOCVD setup for the synthesis of Nb‐doped MoS_2_. A camera image of uniformly grown Nb‐doped MoS_2_ (NbCl_5_ at 55 °C) is shown in the inset. The scale bar is 1 cm. Reproduced with permission.^[^ ^9^ ^]^ Copyright 2020, Wiley‐VCH.b) *I*–*V* characteristics of pristine MoS_2_ and Nb‐doped MoS_2_ with Nb% = 5%. The schematic of the conductive AFM measurements is shown in the inset. Reproduced with permission.^[^ ^9^ ^]^ Copyright 2020, Wiley‐VCH.c) Schematic of type Schottky contact (top) and p‐type Ohmic contact (bottom) explains the observed diode‐Ohmic transition by Nb doping. Reproduced with permission.^[^ ^9^ ^]^ Copyright 2020, Wiley‐VCH. d) Illustration of the vertical, cold‐wall MOCVD system that is used to grow Re‐oped WSe_2_ samples. Reproduced with permission.^[^ ^96^Copyright 2020, Wiley‐VCH.e) Schematic of the multi‐step process showing variation in W(CO)_6_ flow rate that was used to control nucleation, ripening, and lateral growth. Re_2_(CO)_10_ flow is introduced at the lateral growth step. Reproduced with permission.^[^ ^98^ ^]^ Copyright 2018, American Chemical Society. f) The doping data from XPS, SIMS, and extrapolated points are plotted as a function of Re_2_(CO)_10_ flow rate. Reproduced with permission.^[^ ^96^ ^]^ Copyright 2020, Wiley‐VCH.g) Transfer characteristics of 800 °C‐grown pristine and Re‐doped WSe_2_ back‐gated field‐effect‐transistors on 50 nm ALD Al_2_O_3_.While ambipolar conduction in the pristine and lightly doped (0.07% and 0.5%) films is demonstrated, at higher dopant concentrations (1.1%), device performance is degraded due to the loss of semiconducting nature. Reproduced with permission.^[^ ^96^ ^]^ Copyright 2020, Wiley‐VCH. h) Schematic of the MOCVD system for the growth of doped MoS_2_ using gas‐phase precursors. Metering valves are used as the flow controllers for the Nb and Re precursors, while MFCs are used for the other precursors and carrier gases. Reproduced with permission.^[^ ^97^ ^]^ Copyright 2020, American Chemical Society. i) XPS spectra show doping concentrations for different Nb‐MoS_2_ samples. From bottom to top: *C*_Nb_ = 4%, 8%, 11%, and 19%. Reproduced with permission.^[^ ^97^ ^]^ Copyright 2020, American Chemical Society. j) ADF‐TEM image of Nb‐doped MoS_2_ shows misoriented domains in false colors with the corresponding diffraction pattern shown in the inset. The scale bar is 200 nm. Reproduced with permission.^[^ ^97^ ^]^ Copyright 2020, American Chemical Society.k) Semi‐log plot of average *σ*_S_ versus *C*_Nb_. The dash lines represent the theoretical *σ*_S_ assuming full ionization, with mobilities taken to be 10 and 1 cm^2^ (V^−1^ s^−1^) for upper and lower dash lines, respectively. Inset: Band diagram shows the dopant carriers in 2D materials cannot be thermally activated at room temperature due to high activation energy (*kT* shown in light orange). The glow around the cyan circle indicates the a_0_. *E*_V_ represents the valence band, and *E*_I_ represents the dopant energy level. Reproduced with permission.^[^ ^97^ ^]^ Copyright 2020, American Chemical Society.l) Arrhenius plot of devices with *C*_Nb_ = 8%, 11%, and 19% (from bottom to top, respectively). The best fit line for each data set is used to extract *E*_0_. The electrical conductance at various temperatures is normalized to the room‐temperature conductance (*σ*_R_). Reproduced with permission.^[^ ^97^ ^]^ Copyright 2020, American Chemical Society.

Metal‐organic chemical vapor deposition provides a uniform and scalable deposition of 2D TMD films. As one of the standard synthesis tools used in the electronic industry, it provides reliable controllability over the precursors' ratios for synthesizing electronic materials. At an early stage, doping for the carrier‐type modulation of MOCVD‐grown TMDs, like the one demonstrated by Zhang et al., combined powder precursor (NbCl_5_) for dopants (Nb) and gas vapors, including Mo(CO)_6_ and (C_2_H_5_)_2_S, for MOCVD of MoS_2_ (**Figure** **5a**). NbCl_5_ was evaporated at 55 °C using a heating belt and transported into the reactor with carrier gases. In contrast, MO gas vapors were controlled by mass flow controllers (MFCs) and pressure controllers (PCs). The inset image of Figure 5a shows a Nb‐doped MoS_2_ film uniformly grown on a 4 cm^2^ *c*‐plane sapphire. The C‐AFM measurement was performed on both pristine and 5% Nb‐doped MoS_2_ grown on graphene to obtain their *I*–*V* characteristics across the layers (Figure 5b and the schematic setup in inset). The *I*–*V* curve of the pristine MoS_2_ exhibits a Schottky‐diode feature. It shows a high turn‐on voltage at 1 V, which is indicative of the Schottky barrier between the CAFM tip's contact metal (It–Ir with a 5.6 eV work function) and the dominant n‐type carrier of MoS_2_. Conversely, the *I*–*V* curve of the Nb‐doped MoS_2_ film in contact with the AFM tip exhibits high conductivity, implying the Fermi level of the Nb‐doped films has been brought closer to the tip's work function by the p‐type doping. The band alignments before and after Nb‐doping (Figure 5c), constructed with XPS and theory, show the original 1 eV Schottky barrier for electron carriers was reduced, and the Ohmic contact was formed for hole carriers.

Recently, doping TMD crystals by MOCVD has been improved.^[^ ^96^, ^97^ ^]^ Dopants in the range from the parts per million (ppm) to the percentage level can be delivered into the MOCVD reactor through the bubbler manifolds by controlling carrier gas flow. The reactor geometry can be either a vertical cold‐wall reactor (Figure 5d)^[^ ^96^ ^]^ or a horizontal hot‐wall reactor).^[^ ^97^ ^]^ The carbon impurity contamination from either MO precursor (e.g., (CH_4_)_2_Se) or substrate cleanliness in MOCVD‐grown TMD layers has long been a concern. To solve this problem, instead of using CH‐rich MO sources to supply chalcogen, Kozhakhmetov et al. use W(CO)_6_, H_2_Se for MOCVD growth of WSe_2_ and Re_2_(CO)_10_ for supplying Re dopants to grow epitaxial Re‐doped WSe_2_ on sapphire at 700 Torr in a pure H_2_ environment at 800 °C.^[^ ^96^ ^]^ The high dissociation energy of the C—O bond of W(CO)_6_ can reduce carbon contamination.^[^ ^70^ ^]^ The Re dopant concentrations in the grown WSe_2_ layer can be controlled precisely with the Re_2_(CO)_10_ flow. Growing uniform epitaxial TMDs on sapphire via MOCVD requires several steps to establish stable nucleation sites and control steady lateral domain growth,^[^ ^56^, ^98^ ^]^ as described in Figure 5e. When the growth temperature is reached, a high W(CO)_6_ flow is introduced for 2 min to deposit W‐rich WSe*_x_* nanoparticles on sapphire. Then W(CO)_6_ flow will be stopped for a short period of annealing (10 min), promoting the surface ripening of deposited WSe*_x_* nanoparticles into stable nanodomains.^[^ ^98^ ^]^ Next, a lower W(CO)_6_ flow and Re_2_(CO)_10_ are introduced for lateral growth of Re‐doped WSe_2_ domains. If the Re concentration is above 1%, the analysis can be conducted quantitatively with XPS measurement, ideal for large‐area and uniform Re‐doped WSe_2_ films. The Re concentration below the XPS detection limit needs to be confirmed using TOF‐SIMS, which can measure the concentration at the ppm level (Figure 5f). The scanning electron micrograph of 0.5% Re‐doped WSe_2_ shows its uniform coverage, and the Re incorporation does not impact domain alignment compared with an undoped WSe_2_ film (Inset, Figure 5f). The electrical measurement performed on FET made of WSe_2_ with Re concentrations at 0.07%, 0.5%, and 1.1% (Figure 5g) shows the subthreshold slope degrades at 0.5% Re‐WSe_2_, which is the concentration that enables semiconductor‐metallic transition in Re‐doped WSe_2_. Furthermore, the WSe_2_ FET device has a poor on/off ratio and weak gate tunability at 1.1% concentration due to the increased sheet conductivity and increased dopant‐induced impurity scattering.

Gao et al., grew Nb‐MoS_2_ monolayers on fused silica via MOCVD that uses NbCl_5_, Mo(CO)_6_ for Nb and Mo, and DES for S (Figure 5h).^[^ ^97^ ^]^ According to the XPS of Nb binding energy, the Nb concentrations (*C*_Nb_) can be tuned from 4% to 19% (Figure 5i). The polycrystallinity of Nb‐MoS_2_ was confirmed by the selective‐area electron diffraction pattern captured in the TEM experiment (Figure 5j). The electrical measurements indicate that when *C*_Nb_ is at 19% the electrical conductance and mobility of the Nb‐MoS_2_ match the expected values that assume the dopants are fully ionized (Figure 5k). Due to the quantum confinement effect and reduced dielectric screening in 2D MoS_2_, dopant's ionization energy can be as high as 0.4 eV,^[^ ^99^ ^]^ which is much larger than room temperature thermal energy (*k*_B_ *T* is near 26 meV). Therefore, the electrical conduction mechanism in Nb‐MoS_2_ is attributed to thermally activated hopping between localized dopant sites whose electrical conductance (*σ*_S_) can be described using an Arrhenius model, *σ* ≈$\;\mathrm{ex}p^{\frac{-E_{0}}{T}}$, where *E*_0_ is the hopping energy between doping sites and *T* is temperature. *T*‐dependent measurements performed on Nb‐MoS_2_ (Figure 5l) show *E*_0_ decreases from 16, 8, to 3 meV as *C*_Nb_ increases from 8%, 11%, to 19%. Therefore, a high *σ*_S_ of Nb‐MoS_2_ was achieved by degenerately doped impurity band at high *C*_Nb_.

### 4.3 Molecular Beam Epitaxy

Molecular beam epitaxy is a reliable technique to grow high‐quality TMDs thanks to its high purity e‐beam sources and the Knudsen cells and an ultra‐high vacuum (UHV, 10^–10^ Torr) environment that could minimize the impurities in MBE grown films. It is especially beneficial to surface science research because it is typically a part of cluster facilities that combine scanning tunneling microscopy/spectroscopy (STM/STS) and X‐ray photoemission techniques, allowing grown samples to be transferred between an MBE reactor and connected characterization tools without exposing samples to air. MBE's thermodynamic and kinetic aspects for the growth of pristine 2D TMDs had been studied frequently to improve the domain size and coverage of MBE‐grown TMD films limited by the UHV environment and low surface mobility of transition metal elements. MBE for TMDs is mostly done on vdW substrates like graphite or other 2D materials to improve the adatom's surface mobility and minimize unnecessary reactions with substrates. Recently, there are a growing number of studies on doping and alloying TMDs in MBE by taking advantage of its abilities to co‐flow multiple elemental sources and control ratio between the transition metal and chalcogen constituents precisely. Through the cluster facilities combine MBE and characterization, many interesting properties and material chemistry of MBE‐grown doped TMDs can be revealed by XPS and STM/STS measurements.

**Figure 6:**
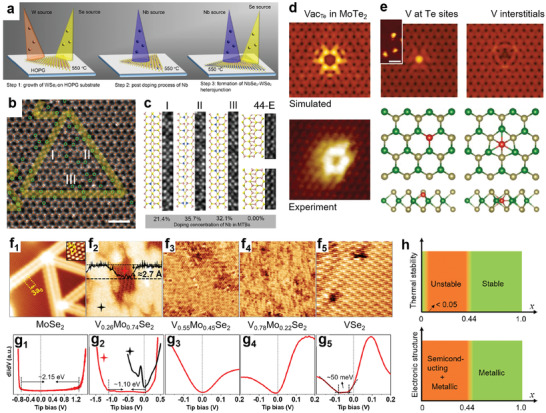
MBE growth of doped and alloyed 2D TMDs. a) MBE growth pathway of Nb doped WSe_2_ at 550 °C. At step 3, niobium sources and selenium sources are introduced to form NbSe_2_ around the outmost Nb‐WSe_2_ and smoothen the surface. Reproduced with permission.^[^ ^100^ ^]^ Copyright 2020, Springer Nature. b) ADF‐STEM image of mirror twin boundary (MTB) loop comprised of three MTBs, I, II, and III. Green circles indicate Nb dopants. The scale bar is 1 nm. Reproduced with permission.^[^ ^100^ ^]^ Copyright 2020, Springer Nature. c) Atomic structure of 4|4‐Se MTBs marked in (b) indicates the local Nb doping concentration inside the boundary. Reproduced with permission.^[^ ^100^ ^]^ Copyright 2020, Springer Nature. d) Simulated STM images and comparison to experimental images for Te vacancy in the negative charge states. Reproduced with permission.^[^ ^101^ ^]^ Copyright 2019, Wiley‐VCH. e) Simulated STM images for V at Te site and V interstitial. Their corresponding atomic structures are shown below. Reproduced with permission.^[^ ^101^ ^]^ Copyright 2019, Wiley‐VCH. f_1–5_) Atomic resolution STM images (15 × 15 nm^2^) of MoSe_2_, V_0.26_Mo_0.74_Se_2_, V_0.55_Mo_0.45_Se_2_, V_0.78_Mo_0.22_Se_2_, and VSe_2_ grown by MBE on graphite. Reproduced with permission.^[^ ^65^ ^]^ Copyright 2020, American Chemical Society. g_1–5_) Corresponding STS curves to each alloy shown in (f). (Set points: f_1_ 1.6 V, 44 pA; f_2_, 1.2 V, 511 pA; f_3_, 0.5 V, 130 pA; f_4_, 0.3 V, 103 pA; and f_5_, −0.02 V, 200 pA.)Reproduced with permission.^[^ ^65^ ^]^ Copyright 2020, American Chemical Society. h) Evolution of (top) thermal stability and (bottom) electronic character of 2D V*_x_*Mo_1−_ *_x_* Se_2_ as a function of *x*. The narrow “stable” region in the top figure means that the thermally stable phase might exist when *x* < 0.05. In (bottom), for *x* ≤ 0.44, the 2D alloys are composed of semiconducting MoSe_2_ domains with metallic MTBs as well as homogeneous, metallic alloy domains. Reproduced with permission.^[^ ^65^ ^]^ Copyright 2020, American Chemical Society.

Wang et al. demonstrated a two‐step MBE to grow Nb‐doped WSe_2_ at 550 °C on graphite.^[^ ^100^ ^]^ WSe_2_ was grown first using e‐beam evaporated W and Se from a Knudsen cell, followed by e‐beam evaporated Nb deposition with a continuous Se supply to incorporate Nb into the existing WSe_2_ lattice (**Figure** **6**a). The authors observed high‐density 1D mirror twin boundaries (MTB) in the WSe_2_ lattice, and Nb dopants mostly exist within 1D MTB instead of distributing homogeneously across WSe_2_ (Figure 6b). The ADF‐STEM image and Z‐contrast intensity of these MTB show substitutional Nb dopants (*C*_Nb_ is 20–30%) are secured inside the MTBs, as shown in Figure 6c. Their DFT calculations indicate that by adding metal atoms into WSe_2_, the formation energy of Nb‐rich MTB will continuously decrease and become a preferable structure to accommodate excess Nb adatoms. A similar case where excess metal‐induced structure transformation was reported on MBE grown Mo‐rich MoSe_2_/MoTe_2_ in which excess Mo are stabilized inside MTB due to its larger lattice spacing.^[^ ^38^ ^]^ MBE has been used to introduce magnetization into 2D crystals by doping magnetic elements. Coelho et al. deposited Vanadium (V) onto MBE‐grown MoTe_2_ at room temperature and found that 0.2% V impurities can raise magnetization in the doped MoTe_2_.^[^ ^101^ ^]^ To understand how V dopants incorporated into the lattice of MoTe_2_, the authors combined STM and image simulation to examine Te vacancies, Te_Vac_ (Figure 6d), V substituted at Te sites (V_Te_), and V interstitials (Figure 6e). While both V_Te_ and V interstitials can raise the magnetic moments in the film theoretically, V_Te_ is mostly responsible for rising the ferromagnetism. It is also consistent with the general concept that impurities could fill in the vacancies without an energy barrier.^[^ ^36^ ^]^

Metal–semiconductor transition in V*_x_*Mo_1‐_ *_x_*Se_2_ alloys was recently explored by a combination of MBE, STM/STS, XPS, and DFT calculations.^[^ ^65^ ^]^ This type of TMD alloys is of great interest for optoelectronic applications because they could provide a large tunable range of bandgap, such as HfS_2_ *_x_*Se_2(1−_ *_x_*_),_ that provide a 0.7 eV tuning range.^[^ ^102^ ^]^ Zhang et al. explored various V*_x_*Mo_1‐_ *_x_*Se_2_ grown by MBE and used STM/STS to understand the evolution of their crystal phases and bandgaps as a function of V concentration *x* (Figure 6f–g). By nature, MoSe_2_ is a semiconductor (see STM/STS in Figure 6f_1_ and g_1_), while VSe_2_ is a metallic material (see STM/STS in Figure 6f_5_ and g_5_). In theory, their combination could lead to a 1.5 eV bandgap tunability.^[^ ^40^ ^]^ The authors found that when *x* is < 0.05, the crystal remains stable and is semiconducting. However, between 0.05 < *x* < 0.44, phase separation occurs, forming interspersed semiconducting and metallic domains (Figure 6f_2_ and g_2_). A similar phase separation issue has been seen on MBE‐grown WSe_2‐_ *_x_*Te*_x_* by Barton et al.,^[^ ^64^ ^]^ in which phase separation occurs with 14% Te incorporation. Once *x* is above 0.44, homogenous stable metallic alloys exist. The thermally stable phase above at *x* ≥ 0.44 was confirmed to be H‐phase. Interestingly, V and Mo strips alternate randomly in parallel stacking to achieve such stability, forming stripe patterns in STM images (Figure 6f_3,4_ and g_3,4_). Their thermal stability and electronic structures as a function of V concentration *x* are summarized in Figure 6h. Despite the challenges for forming continuous, homogenous alloys due to instability at certain V concentration, this study is a good example of using MBE with good stoichiometric control to grow high‐quality TMD alloys used in fundamental research.

### 4.4 Pulsed Laser Deposition

**Figure 7:**
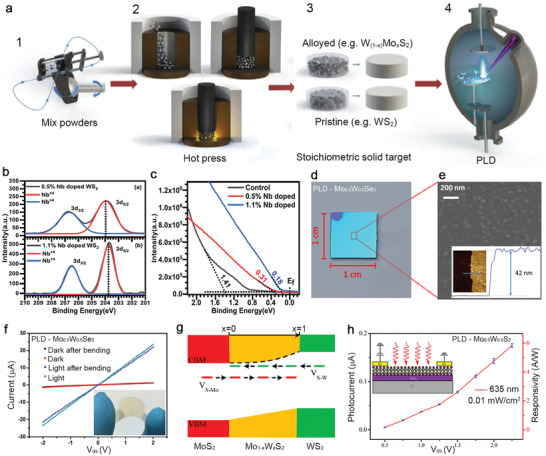
Pulsed laser deposition for doped and alloyed TMDs. a) Illustration of ablation target fabrication and process flow consists of: 1) mixing pristine TMD powders and powders of interest for doping and alloying; 2) hot pressing uniformly mixed powders at elevated temperatures to form; 3) stoichiometric targets for laser ablation; and 4) synthesis of alloyed TMDs films in PLD. Reproduced with permission.^[^ ^105^ ^]^ Copyright 2016, American Chemical Society. b) Nb 3d XPS spectra of the 0.5 and 1.1 atomic percentage (at%) Nb‐doped WS_2_ films grown by PLD. Reproduced with permission.^[^ ^106^ ^]^ Copyright 2018, AIP Publishing. c) Valence band edge of undoped WS_2_, and 0.5 and 1.1 at% Nb‐doped WS_2_ film with respect to Fermi level indicates the Fermi level shifts toward the valance band edge with the increased Nb incorporation. Reproduced with permission.^[^ ^106^ ^]^ Copyright 2018, AIP Publishing. d) Camera image of a PLD‐grown Mo_0.5_W_0.5_Se_2_ alloy film on 1 cm^2^ SiO_2_/Si. The top left corner of the substrate provides the contrast of bare silica. Reproduced with permission.^[^ ^107^ ^]^ Copyright 2017, American Chemical Society. e) The scanning electron micrograph of an as‐grown Mo_0.5_W_0.5_Se_2_ film provides a close view of compact nanodomains on the surface. The inset shows the thickness of the same film. Reproduced with permission.^[^ ^107^ ^]^ Copyright 2017, American Chemical Society. f) *I*−*V* characteristics of the flexible two‐terminal device are acquired in the dark and under light illumination. Inset shows a camera image of a flexible Mo_0.5_W_0.5_Se_2_ alloy film under bending deposited on a PI substrate. Reproduced with permission.^[^ ^107^ ^]^ Copyright 2017, American Chemical Society. g) Illustration of the energy band diagram for Mo _1−_ *_x_*W*_x_*S_2_ alloys as a function of *x*. Reproduced with permission.^[^ ^108^ ^]^ Copyright 2016, American Chemical Society.h) Voltage‐dependent photocurrent and responsivity of the Mo _1−_ *_x_*W*_x_*S_2_ detector under the illumination of a 635 nm laser. Inset is a schematic of the photodetector. Reproduced with permission.^[^ ^108^ ^]^ Copyright 2016, American Chemical Society.

Pulsed laser deposition is a versatile, highly scalable physical vapor deposition technique to grow functional nanomaterials.^[^ ^52^ ^]^ The laser vaporization of an ablation target by a high‐power pulsed laser (most frequently used wavelength: 248 nm, pulse width: 25 ns) can create plasma plumes that deliver the stoichiometric precursors from the ablation target onto a substrate.^[^ ^103^ ^]^ Therefore, PLD has been widely used to grow 2D materials and is especially suitable for the growth of multi‐composition alloyed or doped 2D materials. Compared to other growth techniques, PLD could grow TMDs at a lower temperature because the deposited molecules have high kinetic energies that could promote their surface diffusion or heat the substrate locally upon deposition.^[^ ^104^ ^]^ There are usually two ways to dope materials by PLD through mixing the dopants either to the target or into the background gas, but the latter one is often used for doping nitrogen or oxygen and other non‐metal elements. The ablation target's composition can be carefully controlled by mixing a specific ratio of pristine TMDs and dopants into a densified target to grow a variety of TMD alloys (**Figure** **7**a). Typically, extra chalcogen powders such as Se and S will be added to targets to compensate for their rapid loss during high‐temperature synthesis due to their high vapor pressure.^[^ ^105^ ^]^ Rathod et al. demonstrated PLD‐grown Nb‐doped WS_2_ films with controllable Nb concentrations using ablation targets fabricated from the mixture of WS_2_, S, and Nb powders. The XPS experiment confirmed the compositions in as‐grown Nb doped WS_2_ are close to stoichiometry, especially the improved W/S ratios due to the extra sulfur in the target (Figure 7b).^[^ ^106^ ^]^ Ultraviolet photoemission spectroscopy was used to determine the Fermi level position from the valence band (VB) edges of the undoped and doped films. The Fermi level of PLD‐grown undoped WS_2_ film is at 1.41 eV from its VB edge. Conversely, the Fermi levels of Nb‐doped WS_2_ films with 0.5 and 1.1 Nb% are at 0.31 and 0.18 eV from their VB edge (Figure 7c). They found that the Nb doping in WS_2_ could effectively switch the n‐type conductivity in pristine WS_2_ to p‐type conductivity and induce enhanced mobility up to 7.2 cm^2^ V^−1^ s^−1^. This work demonstrates that PLD is an efficient way for controlled doping or alloying of 2D TMDs.

Yang et al. recently demonstrated the growth of uniform W_0.5_Mo_0.5_S_2_ and W_0.5_Mo_0.5_Se_2_ alloy films grown by PLD (Figure 7d).^[^ ^107^, ^108^ ^]^ The ablation targets for these ternary alloys were made of either the 1:1 mixture WSe_2_ and MoSe_2_ powders for W_0.5_Mo_0.5_Se_2_ or the mixture of Mo, W, and S powders (1:1:4) for W_0.5_Mo_0.5_S_2_. With laser ablation of the target, the high‐quality alloy films with the right stoichiometry were fabricated at the centimeter‐scale on SiO_2_ substrate. (Figure 7e).^[^ ^107^ ^]^ Due to the low growth temperature at 400 °C, the W_0.5_Mo_0.5_Se_2_ alloy was also directly grown on a polyimide (PI) substrate for a flexible and transparent photodetector (Figure 7f). PLD‐grown film's photoresponse on flexible PI substrates only changes a little after bending 100 times (the flexed state in inset, Figure 7f), indicating robust mechanical flexibility and high quality of as‐grown alloy films.^[^ ^108^ ^]^ Such superior photodetector performance is brilliantly achieved on PLD‐grown alloy films by engineering the alloy compositions to minimize defect‐assisted recombination process (Figure 7g) and increase the thermal stability of alloys and the photodetector performance (Figure 7h). These examples demonstrate the versatility for synthesizing doped and alloyed TMDs in PLD simply by tuning ablation targets' composition. Additionally, these examples prove that alloying is effective for improving material quality and device performance of PLD grown TMD films.

### 4.5 Low‐Temperature Efforts

The development of scalable, low‐temperature synthesis methods for high‐quality doped and alloyed 2D TMDs is necessary for their integration in many applications such as Si CMOS technology at the BEOL level^[^ ^109^, ^110^ ^]^ and flexible electronics.^[^ ^111^, ^112^ ^]^ Substrates for these technologies are amorphous and can only sustain limited thermal stress (<500 °C) before they degrade or damage neighboring layers. The majority of large‐scale TMD synthesis, however, has been demonstrated at high temperatures (>700 °C) and on single‐crystalline substrates through CVD_SS_ or MOCVD.^[^ ^30^, ^56^ ^]^ In vapor‐phase synthesis, high growth temperatures result in films with improved crystallinity and larger grain sizes due to the lower nucleation events and higher adatom surface diffusion than the films grown at low temperatures.^[^ ^61^ ^]^ At low growth temperatures, the growth shifts from a mass‐transfer limited process to a surface‐reaction controlled process^[^ ^62^ ^]^ with higher attachment rates and lower surface adatom mobility yielding smaller, less geometrically “sharp” grains.^[^ ^61^ ^]^ Efforts in obtaining higher‐quality TMD films at lower growth temperatures include growth promoters,^[^ ^60^, ^113^ ^]^ substrate engineering,^[^ ^114^ ^]^ or careful precursor ratio tuning.^[^ ^68^, ^73^, ^115^ ^]^ Solution‐based methods have also been explored to produce 2D TMDs at low processing temperatures.^[^ ^116^, ^117^ ^]^ While TMD synthesis on substrates with low thermal budgets is gaining traction, the development of doping strategies for bottom–up approaches at low growth temperatures is still in its infancy.

**Figure 8:**
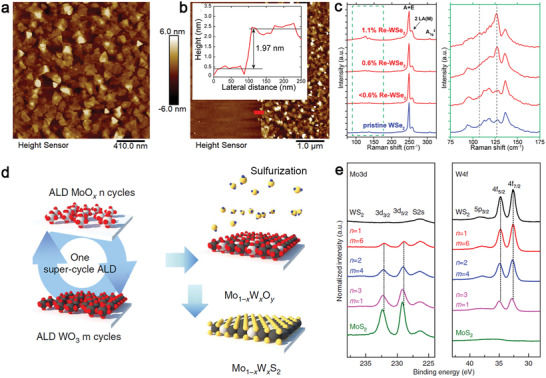
Low‐temperature synthesis of doped and alloyed TMDs. a,b) AFM images demonstrate that Re doped WSe_2_ films grown on SiO_2_/Si substrates at 450 °C by MOCVD are polycrystalline and have domain sizes about 150–200 nm with an average thickness of 2 nm, corresponding to 3–4 layers. c) Raman spectra of pristine and Re‐doped WSe_2_ analyzed by 532 nm excitation wavelength. The intensity of ZA(M) and LA(M) modes around 146 cm^−1^ in both cases are enhanced as a function of Re concentration. (a‐c): Reproduced with permission.^[^ ^96^ ^]^ Copyright 2020, Wiley‐VCH. d) A synthesis flow of super‐cycle ALD for Mo_1‐_ *_x_*W*_x_*O alloy and sulfurization of H_2_S for Mo_1‐_ *_x_*W*_x_*S_2_. e) XPS measurements for Mo 3d and W 4f core levels in the Mo_1‐_ *_x_*W*_x_*S_2_ alloy with different *n* and *m* numbers in one super‐cycle. (d‐e): Reproduced under the terms of the CC‐BY 4.0 license.^[^ ^121^ ^]^ Copyright 2015, The Authors, published by Springer Nature.

For vapor phase synthesis, an appropriate precursor must be identified to enable controllable doping at low temperatures. While CVD_SS_ has been widely employed to demonstrate TMD doping at relatively high growth temperatures, their applications to substrates with a limited thermal budget could be problematic. Metal oxide powders that are ubiquitous in powder‐based CVD_SS_, in‐fact, break down at temperatures far beyond the BEOL limit (<700 °C). MOCVD does not suffer from this limitation, however. There is an extensive library of metal‐organic precursors that undergo clean pyrolysis at relatively low temperatures.^[^ ^118^ ^]^ Furthermore, by placing precursors inside of pressure and temperature regulated bubbler, MOCVD offers much higher control on precursor delivery than the powder‐based CVD approach making it a perfect candidate for achieving controllable substitutional doping at low growth temperatures. Kozhakhmetov et al. flowed H_2_ carrier gas through a Re_2_(CO)_10_ bubbler during MCOVD WSe_2_ synthesis to demonstrate Re's successful incorporation in the host lattice at 450 °C on SiO_2_/Si with tunable doping concentration.^[^ ^96^ ^]^ These precursors, including W(CO)_6_, H_2_Se, and Re_2_(CO)_10,_ can dissociate at relatively low temperatures below the BEOL limit.^[^ ^51^ ^]^ The Re‐doped WSe_2_ films are polycrystalline on SiO_2_/Si and are three‐layer thick (**Figure** **8a**,b). Raman spectra of Re‐doped WSe_2_ films with the increased concentration (<0.6%, 0.6%, and 1.1%) retain the primary phonon modes of pristine WSe_2_ at 251 (A + E) and 260 cm^−1^ (2LA(M)) (Figure 8c). Additionally, the defect‐activated modes at around 126 cm^−1^ become more intense and narrower along with the increased concentration.

Atomic layer deposition is a self‐limited deposition technique frequently used for low‐temperature conformal coating of amorphous oxides like TiO_2_ and Al_2_O_3_ desirable as the high‐*k* dielectric layer for FET. ALD can directly grow TMD films with excellent uniformity, incomparable conformity, and nanoscale controllability.^[^ ^119^ ^]^ Additionally, some of ALD‐grown transition metal oxides (TMO) such as MoO*_x_* and WO*_x_* can be integrated with post‐deposition chemical vapor treatment to grow TMDs.^[^ ^120^ ^]^ Its growth mechanism proceeds through four steps: the precursor/purge/purge pulse of co‐reactant/purge. Each precursor and co‐reactant's exposure adjusted to ensure its surface reaction is saturated before the next starts. The deposited surface needs to have dangling bonds or be reactive to initiate the reaction. Although the precursors comprised of TM elements such as WF_6_, MoCl_5_, Mo(CO)_6_ can react below 100 °C, the actual growth temperatures of ALD‐deposited TMD films need to be annealed at higher temperature to transform them from amorphous or nanocrystalline into crystalline films for most practical applications. High‐quality W_(1‐_ *_x_*_)_Mo*_x_*S_2_ monolayer and few‐layer alloys have been demonstrated by a two‐step synthesis consisting of ALD and post‐growth chemical treatment.^[^ ^121^ ^]^ In that work, arbitrary cycles of ALD MoO*_x_* and WO_3_ are deposited successively at 200 °C to create W_(1‐_ *_x_*_)_Mo*_x_*O (Figure 8d). Subsequently, the TMD alloys are sulfurized into W_(1‐_ *_x_*_)_Mo*_x_*S_2_ using H_2_S at a higher temperature. The XPS experiments indicate that the atomic ratios between Mo and W can be tuned by changing the cycling number of MoO*_x_* (*m*) and WO_3_ (*n*) before high‐temperature, vapor‐phase chalcogenization (Figure 8d). Although high‐temperature annealing (>700 °C) is inevitably required to achieve high‐quality TMD alloys deposited by ALD processes, it is still promising for the BEOL process and can use laser annealing to perform chalcogenization at room temperature.

Molecular beam epitaxy is also a promising route to achieve doped‐TMD at relatively low growth temperatures due to its vast range of possible dopant precursors and its unmatched control over precursor flux to the growth substrate. Furthermore, unintentional doping of layers is highly unlikely due to the UHV growth environment. Wang et al. demonstrated controllable Nb doping of MBE‐grown WSe_2_ on graphite.^[^ ^100^ ^]^ While this study's growth temperature for Nb‐doped WSe_2_ was 550 °C, the MBE growth of TMDs has been demonstrated at even lower growth temperatures.^[^ ^122^, ^123^, ^124^ ^]^

## 5 Top–Down Approaches

### 5.1 Low Energy Ion Implantation

Besides synthetic doping methods, post‐growth, top–down processing methods also enable controlled doping of foreign atoms into 2D TMDs. It is typically carried out in CVD_SS_‐grown samples at high temperature that facilitates the ion‐exchange between vapor phase dopants and the constituents of 2D TMDs. High temperature is required to meet the displacement threshold energy (*T*_d_), which is the minimum energy that one constituent atom needs to desorb and form a defect. For example, 6–8 eV per atom is required for desorbing the chalcogen and 20–30 eV per atom for the transition metal in 2D TMDs. In other words, it requires the temperature to be more than 700 °C to fulfill 6–8 eV per atom for *T*_d_ of the chalcogen. High temperature processing for doping 2D materials is not ideal because it causes a large volume of defects in 2D crystals and induces the strain to crack the crystal or form the wrinkles, therefore significantly degrading the crystal quality.

**Figure 9:**
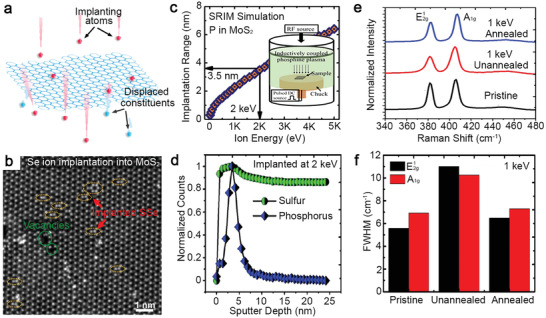
Ion implantation for doping 2D TMDs. a) Schematic of energetic implanting dopants (red) that sputter of the constituents of 2D materials and fill in the vacancies to complete substitutional doping. Reproduced with permission.^[^ ^125^ ^]^ Copyright 2013, American Chemical Society. b) Gaussian‐filtered ADF‐STEM image of Se ions‐implanted MoS_2_ monolayer. Orange and green rings are SSe pairs and vacancies created by Se implantation. Reproduced with permission.^[^ ^79^ ^]^ Copyright 2016, Elsevier. c) SRIM simulation data shows the implantation range of P in MoS_2_ for increasing ion energy in the PIII process. d) SIMS profiles obtained from P‐implanted MoS_2_ show shallow phosphorus doping with a sharp Gaussian‐like distribution compared to background S. e) Raman spectra and f) FWHM values of Raman peaks (*E* ^1^_2g_ and *A*_1g_) for pristine, implanted by P at 1 keV (unannealed), and post‐implantation annealed MoS_2_. (c‐f): Reproduced with permission.^[^ ^12^ ^]^ Copyright 2016, American Chemical Society.

Ion implantation is an efficient and economical way for doping. It is already a CMOS compatible semiconducting technique to achieve small‐depth channel doping on Si using energetic ions. However, the high energy beams in traditional ion implantation techniques will lead to significant crystal damage for 2D materials. Therefore, it is important to develop low‐energy ion implantation techniques to perform implantation with ion energies down to 10 eV in a controlled and localized way that is very suitable for 2D materials. For implanting 2D materials, energetic ions serve two roles. First, they will sputter off the constituents by physical collisions and create single vacancies; Second, once they are stopped on the 2D surface after losing their kinetic energy in the collisions, they can fill in the vacancies, completing the substitutional doping (**Figure** **9a**).^[^ ^125^ ^]^ Previous research on doping graphene with B and N with ion implantation has suggested that ion irradiation's energy is slightly lower than the threshold for single vacancies to reduce large‐size defects but increase the substitution‐to‐defect ratio.^[^ ^125^ ^]^ The same principles can be applied to monolayer TMDs. Bangert et al. demonstrated Se implantation on 2D MoS_2_ that substitutes S with Se ions with 10 eV per atom kinetic energy. This value is comparable with the displacement energy value of S in MoS_2_ (7–10 eV per S).^[^ ^36^ ^]^ ADF‐STEM image of Se ion‐irradiated suspended MoS_2_ monolayer shows a low density S sites have been substituted with Se that were marked with yellow circles (Figure 9b). However, large vacancies might have also been created during Se ion irradiation (green circles in Figure 9b), indicating the kinetic energy could be reduced further to prevent large‐size vacancy formation.

Another technique that can provide low‐energy implantation for small‐depth doping is a plasma immersion ion implantation (PIII). In a demonstration by Nipane et al.,^[^ ^12^ ^]^ an inductively coupled plasma source creates low energy high‐density P plasma that is regulated by an opposite DC bias between 0 and 2 keV to reduce the plasma density and also etching rate near sample surface (Figure 9c). The authors used the stopping‐range of ions in matter (SRIM) simulations to estimate P implantation depths in MoS_2_ for increasing implantation energies. The SRIM simulations and experimental second ion mass spectrometry (SIMS) performed on an implanted MoS_2_ crystal confirmed that PIII at 2 keV would make an implantation range of 3.6 nm, which is equivalent to the top 5–6 layers of MoS_2_ (Figure 9d). However, PIII implantation would also induce lattice distortion and defects in 2D crystals. To understand implantation‐induced structural distortions, the authors used Raman spectra of MoS_2_ as indicators to monitor MoS_2_ crystals’ quality before and after implantation at 1 keV bias and after post‐implantation annealing at 300 °C (Figure 9e,f). After implantation, both *E* ^1^_2g_ and *A*_1g_ peaks showed full‐width‐half‐maximum (FWHM) broadening by 4 cm^−1^, implying the sample had been damaged. Annealing at 300 °C subsequently recovers the FWHM for both Raman peaks close to values for a pristine sample. This combined implantation and annealing approach is analog to standard implantation for doping Si‐based electronics. PIII has been used to achieve p‐type carrier modulation on n‐type MoS_2_ by shallowing implanting P in the top few layers of MoS_2_.

**Figure 10:**
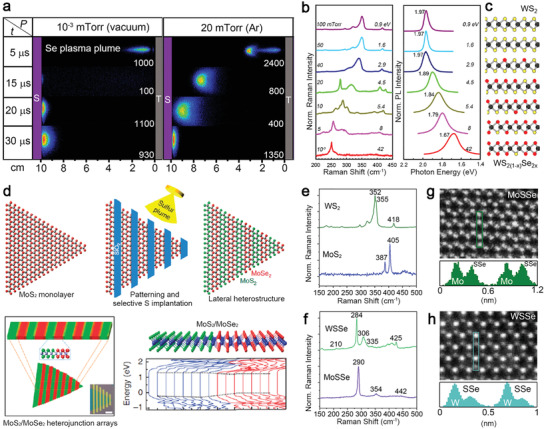
Hyperthermal energy implantation for doping 2D materials by PLD. a) False color, gated‐ICCD images of the Se plasma plume's visible luminescence reveal the plume's propagation dynamics through the vacuum and 20 mTorr Ar background gas pressures at the indicated delay times following the laser pulse. (Gate width is 10% of each delay time. The maximum intensity is shown for comparison.) b) Raman spectra of WS_2_ monolayers on SiO_2_/Si substrates (left) and their corresponding PL spectra and peak energy positions (right) exposed by 800 Se plasma plume pulses in different Ar background gas pressures and plume kinetic energies at 250 °C. Spectra indicate an increasing fraction of Se incorporation in WS_2(1−_ *_x_*_)_Se_2_ *_x_* with decreasing Ar pressure. c) Schematic structural evolution of Se incorporation in W_S2(1−_ *_x_*_)_Se_2_ *_x_* corresponding to the Raman/PL spectra in (b). (a‐c): Reproduced with permission.^[^ ^67^ ^]^ Copyright 2020, American Chemical Society. d) Schematic for the formation of lateral MoSe_2_/MoS_2_ heterojunction arrays within a monolayer by patterning with SiO_2_ hard masks and selective conversion using S plasma plume (top row). Green, red, and combined Raman maps were obtained from corresponding optical images representing MoS_2_ and MoSe_2_ at 403 and 238 cm^−1^. Scale bar is 5 µm. First‐principles calculations of a lateral MoSe_2_/MoS_2_ junction show formation of type‐I band alignment. Reproduced under the terms of the CC‐BY 4.0 license.^[^ ^126^ ^]^ Copyright 2015, The Authors, published by Springer Nature. e) Raman spectra of as‐grown CVD MoS_2_ and WS_2_ monolayers. f) Raman spectra of the Janus MoSSe and WSSe monolayers converted from the same samples are shown in (e). g,h) ADF‐STEM image of MoSSe (g) and WSSe (h) and the Z‐contrast intensity profiles in the highlighted sections in the images verify the Janus structure. (e‐h): Reproduced with permission.^[^ ^67^ ^]^ Copyright 2020, American Chemical Society.

Laser‐ablation plumes that are typically generated for film deposition in PLD provide an intriguing new opportunity for implanting dopants.^[^ ^67^, ^126^ ^]^ Although the effects of kinetic energy are often overlooked in film deposition, at the monolayer or few‐layer level of 2D TMDs it becomes apparent that the natural hyperthermal KE acquired in the free expansion of laser‐plasma plumes in vacuum, although typically less than 100 eV per atom, is sufficient to implant or sputter 2D layers. In fact, recent work has shown that it is necessary to reduce the natural KEs acquired by the atoms, ions, and clusters that freely expand from a laser‐ablated target when they are used as irradiating dopants to prevent irreparable defects in 2D TMD monolayers. Thus, compared to standard ion implantation where the KE is typically above 100 eV per atom, the KE of laser‐plasma plumes is naturally much better suited to direct implantation of dopants. Moreover, the method is extremely versatile for generating pure beams of virtually any material simply by laser ablation. Using inert background gases between the target and the substrate, the plume atoms can be controllably slowed in accordance with scattering models. In this way, the maximum KE of species arriving at the substrate is smoothly tunable in PLD. Therefore, by controlling Se species using an Ar background, KE's in the <5 eV per atom range can be gently tuned to selectively convert a single MoS_2_ or WS_2_ monolayer to implant just one side, making novel Janus monolayers of MoSSe or WSSe with the different chalcogens on either side. The background gas pressure‐controlled KE reduction can be understood by comparing sequences of gated intensified CCD (ICCD) camera images of the plume propagation captured with (20 mTorr) and without (10^−6^ Torr) the Ar background at 5, 15, 20, and 30 µs after a single pulse of laser ablation (**Figure** **10a**). In the ICCD images captured at 20 µs, Se plumes can be seen to have already landed on the substrate surface in vacuum, while they are still traveling at 20 mTorr, indicating that gas collisions had decelerated the plumes. By simply adjusting the background gas pressure, the high KE of atoms and clusters embedded in plasma plumes generated in vacuum can be controllably reduced using Ar gas collisions from originally 40 eV per atom to achieve just 3–5 eV per atom. The effects of this KE reduction of the Se plumes by the background pressure in PLD is seen to control the Se/S ratio in WS_2(1‐_ *_x_*_)_Se_2_ *_x_* alloys and also the implantation range, as revealed by the evolution of the associated Raman and PL spectra of the resulting WS_2(1‐_ *_x_*_)_Se_2_ *_x_* alloys as depicted in Figure 10b,c, respectively. With the higher kinetic energies, both sides of a monolayer can be implanted and converted fully from sulfides to selenides, or selenides to sulfides.

Using this technique and pattern masks, Mahjouri‐Samani et al. created multiple lateral heterojunctions of MoS_2_‐MoSe_2_ by selectively converting MoSe_2_ to MoS_2_ with energetic S plasma plumes (40 eV per atom) generated by laser ablation of a sulfur target in vacuum for PLD (Figure 10d).^[^ ^126^ ^]^ The areas protected by patterned SiO_2_ hard masks remained MoSe_2,_ while unprotected areas were converted to MoS_2_ by S species traveling with kinetic energies up to 40 eV per atom. Besides implantation to create homogeneous alloys or full S‐Se conversion of 2D layers, Janus TMD monolayers such as MoSSe and WSSe were achieved by selective selenization with Se plasma plumes. Lin et al. tuned the KE of Se plasma plumes down to a narrow 3–5 eV per atom range to selectively implant Se into only the top‐most S layer of CVD‐grown MoS_2_ and WS_2_ monolayers for making their Janus structures at 300 °C.^[^ ^67^ ^]^ The Raman spectra show the monolayers' phonon signatures before (Figure 10e) and after (Figure 10f) the Janus conversion, matching theoretical predictions. ADF‐STEM images in tilted geometry of the resulting Janus MoSSe and WSSe monolayers confirmed that the S‐Se pairs were consistently ordered with all the Se atoms within the top‐most layer. Compared to other methods for producing 2D Janus structures such as high‐temperature ion exchange and plasma‐assisted conversion, this low energy implantation method can selectively produce high‐quality 2D Janus layers alloys through the process of repeated Se implantation to cause Se‐rich nanoscale domains, followed by recrystallization, which leads to selective ejection of S species.

### 5.2 Doping and Alloying through Artificial Defects Created by Energetic Ions

**Figure 11:**
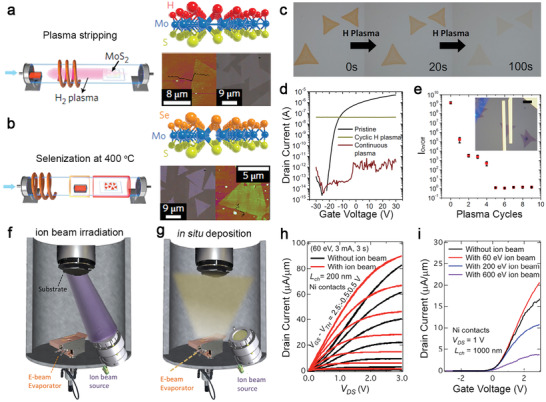
Modifying compositions and structures in 2D semiconductors through vacancies. a) A MoS_2_ monolayer was exposed to H_2_ plasma to strip the top‐layer S. The plasma was then switched off, and Se powder loaded in a quartz boat was moved next to the sample without breaking the vacuum. b) Se was then thermally vaporized to achieve selenization and complete the synthesis of Janus MoSSe. Optical micrograph and AFM images for each structure are shown below the molecular model for each stage. (a‐b): Reproduced with permission.^[^ ^88^ ^]^ Copyright 2020, AIP Publishing. c) Optical micrographs of pristine MoS_2_ and after various H_2_ plasma exposure time. d) Transfer curves for pristine and cyclic and continuous H_2_ plasma exposed MoS_2_. e) On/off ratio as a function of the cycles of “10 s plasma + atmosphere exposure”. An optical micrograph of the tested FETs is shown in the inset. The scale bar is 5 µm. (c‐e): Reproduced under the terms of CC‐BY 4.0 license.^[^ ^129^ ^]^ Copyright 2019, The Authors, published by Springer Nature. f) Experimental setup of the in situ convergent Ar+ ion beam source with an e‐beam evaporator within an ultra‐high vacuum (UHV) chamber. The ion beam irradiation first creates defects in samples. g) In situ Ni evaporation process was carried out right after ion beam exposure. (f‐g): Reproduced with permission.^[^ ^89^ ^]^ Copyright 2019, American Chemical Society. h) Comparison of the output characteristics of the FET fabricated on both irradiated and pristine MoS_2_ with Ni contact. i) Comparison of the effect of different ion beam energies on device transfer characteristics. The ion beam exposure conditions are (60 eV, 3 mA, 3 s), (200 eV, 3 mA, 3 s), and (600 eV, 36 mA, 3 s), respectively. (h‐i): Reproduced with permission.^[^ ^136^ ^]^ Copyright 2020, Royal Society of Chemistry.

The presence of defects can significantly reduce *E*_f_ of incorporating a substitutional dopant into a 2D TMDs.^[^ ^36^ ^]^ Theoretical studies for point‐defect formation in 2D TMDs indicate the difference in the displacement thresholds (*T*_d_) for TM and chalcogen constituent is significantly large because six covalent bonds secure a single TM in 1H‐TMD while a single chalcogen is only secured by three. The *T*_d_ for Mo and S in a MoS_2_ monolayer is 30 and 6 eV,^[^ ^127^ ^]^ respectively. Therefore, compared to Mo, it is easier to remove S to provide vacancies for hosting dopants. Lu et al. used H_2_ plasma treatment to strip the top S atoms of a 2D MoS_2_ using an inductively coupled plasma system.^[^ ^80^ ^]^ H_2_ plasma was ignited with a 50 W radio‐frequency power source and operated at 100 mTorr chamber pressure. Ionized H_2_ molecules were believed to react with S atoms and remove them by forming HS* radicals or H_2_S. The authors hypothesized that the S vacancies would be passivated by atomic hydrogen after the first stripping step (**Figure** **11a**). Subsequently, vaporized Se was introduced to fill in the top S sites and complete a Janus MoSSe when the furnace was 400 °C (Figure 11b). The authors showed that after the two‐step conversion, the surface of Janus MoSSe has cracks and tears, possibly due to the release of compressive strain. Nevertheless, this method is promising for very low‐temperature Janus conversion. Trivedi et al. demonstrate a single‐step H_2_ plasma treatment that converts 2D MoSe_2_ into 2D Janus MoSeS at room temperature.^[^ ^128^ ^]^ The H_2_ plasma strips off the top Se layer and converts loaded S powders into H_2_S vapor that fills in Se vacancies simultaneously.

Stanford et al. used a similar approach with H_2_ plasma to controllably place oxygen atoms into existing MoS_2_ monolayers.^[^ ^129^ ^]^ First, the authors demonstrated that increasing H_2_ plasma treatment time between 0 and 100 s only gradually removed MoS_2_ (Figure 11c). A combination of H_2_ plasma and O_2_ plasma was used to create point defects in MoS_2_ with the first treatment and fill in atomic oxygen into the defects with the second treatment. The transfer characteristics of FET fabricated on 1) pristine, 2) H_2_ plasma‐treated, and 3) H_2_/O_2_ plasma‐treated MoS_2_ monolayers indicate that the H_2_/O_2_ plasma treatment makes MoS_2_ metallic because of the MoO*_x_* incorporation (Figure 11d). Cycle numbers of the combined treatment controlled the amount of oxygen incorporation. For example, the on/off ratio of the transfer characteristic from the same device can be controllably reduced by sequential cycles of the combination of H_2_ plasma treatment and ambient exposure (Figure 11e).

In addition to the doping and etching processes for semiconductor applications, ion beams, and cluster irradiation are promising for the defect engineering of 2D materials to alter their mechanical, electrical, and optical properties.^[^ ^130^, ^131^, ^132^, ^133^ ^]^ Examples include the phase transformation of 2D MoS_2_ from semiconducting to metallic phase by Ar^+^ plasma,^[^ ^134^ ^]^ and defect‐induced carrier modulation demonstrated on 2D TMDs using a focused He^+^ ion beam.^[^ ^131^ ^]^ It is essential to control the beam energy and ion flux to create point defects without etching away one layer. Ideally, after defects are made, in situ dopants should be introduced in a high vacuum to minimize oxidation and unwanted contamination caused by air exposure. Zheng et al. used a convergent ion Ar^+^ beam and an in situ e‐beam evaporator integrated into a vacuum chamber.^[^ ^54^ ^]^ The authors found that a convergent ion beam source can reduce impurities in irradiated crystals compared to a traditional broad ion beam source. The convergent ion beam is directional and can avoid sputtering the chamber inner wall coating. The Ar^+^ ion beam source irradiates MoS_2_ samples with tunable ion energy and flux at 10^−4^ Torr (Figure 11f), followed by deposition of metals or dopants using an in situ e‐beam evaporator (Figure 11g). The authors applied 60 eV ion beam irradiation for 3 s to modify the contact area of MoS_2_ FETs before Ni deposition and compared it to FETs without ion beam irradiation. Compared to the unmodified FETs, the output characteristics of the modified FETs are improved in the low source‐drain voltage (*V*_DS_) region (<0.5 V), evident by larger drain current and smaller contact resistance (Figure 11h). It is possible that Ni reacts with defects and distorted lattices induced by ion beam and form bonds, thereby improving the carrier injection.^[^ ^135^ ^]^ FETs' transfer characteristics modified by 200 and 600 eV ion beam exposure only show degradation in contact performance due to excessive damage and disorder created by the higher energy ions (Figure 11i). Although this work did not directly exploit this approach to dope TMDs, its controllable ion beam energy and in situ evaporation can be applied to compositional engineering.

### 5.3 Laser‐Assisted Modifications and Growth

**Figure 12:**
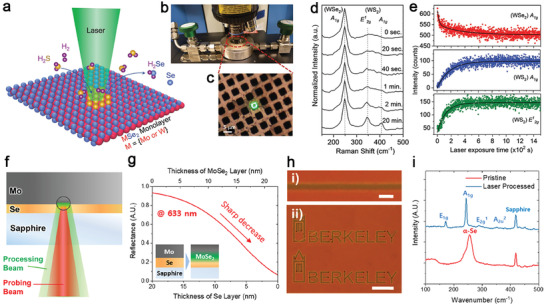
Laser‐assisted modifications and growth of TMD crystals. a) A schematic of the laser‐assisted S‐Se conversion in an H_2_S atmosphere. b) Image of the mini environmental chamber under the microscope‐based laser spectrometer. c) The laser beam (green) focused on a WSe_2_ monolayer suspended on a TEM grid. d) Time‐dependent evolution of the Raman spectra for the WSe_2_ (*A*_1g_) and WS_2_ (*A*_1g_ and *E*_2g_) under laser irradiation (532 nm, 0.7 mW) in an H_2_S atmosphere. e) Time‐dependent evolution of the Raman peak intensity. (a‐e): Reproduced with permission.^[^ ^82^ ^]^ Copyright 2018, Wiley‐VCH. f) A schematic of the laser‐assisted growth ofMoSe_2_ from the sapphire/Se/Mo multilayer stack. In situ reflectance measurement showing the processing time for the saturation of MoSe_2_ growth. g) Calculated reflectance of the Se/MoSe_2_/Mo multilayer at a 633 nm laser beam incident from the sapphire side as a function of the Se layer's thicknesses and the MoSe_2_ layer formed. The laser beam was irradiated through the transparent sapphire substrate, and MoSe_2_ was formed at Se/Mo interface (inset). h) Optical micrographs obtained through the sapphire substrate show laser‐written (i) simple line and (ii) arbitrary patterns. The scale bars in panels (i) and (ii) are 10 and 100 µm, respectively. (i) Raman spectra obtained from the pristine and laser‐processed area. (f‐i): Reproduced with permission.^[^ ^84^ ^]^ Copyright 2020, American Chemical Society.

Laser‐assisted synthesis and processing have emerged as a reliable and controllable technique for spatial tailoring of material properties. Direct laser writing can be used to grow graphene on various surfaces that contain carbon and also TMD crystals.^[^ ^137^, ^138^ ^]^ Its scanning rate and laser power can be tuned to create various metastable phases and compositions. For post‐growth modifications, laser irradiation can be applied to thin down layered crystals down to single‐layer,^[^ ^139^ ^]^ heal Se vacancies in WSe_2_ by promoting oxygen incorporation under the ambient conditions,^[^ ^140^ ^]^ pattern devices directly on 2D films for applications,^[^ ^141^ ^]^ and enable site‐specific phosphorous doping of MoS_2_ and WSe_2_ in an environmental chamber,^[^ ^13^ ^]^ to name a few. A laser‐assisted process can combine in situ laser spectroscopy to capture Raman's dynamic evolution and photoluminescence of modified 2D crystals on the fly to understand laser‐crystallization kinetics or photochemical mechanisms in the process.^[^ ^82^, ^142^, ^143^ ^]^ Afaneh et al. provide a great example of using laser processes and laser spectroscopy to engineer 2D alloys and elucidate the conversion mechanisms all together in a single‐step experiment.^[^ ^82^ ^]^ The authors demonstrated S–Se conversion on a suspended WSe_2_ monolayer in an H_2_S environment with a combination of laser annealing and spectrometer (**Figure** **12a**–c). Typically, the S‐Se conversion is a thermal process conducted in a tube furnace loaded with chalcogen powders. While the thermal conversion requires high‐temperature annealing, the reported laser‐assisted conversion can be performed at room temperature with a 532 nm laser.^[^ ^82^ ^]^ The authors found that the photochemical conversion between H_2_S and WS_2_ can be triggered with 0.7 mW, which is adequate to generate Se vacancies that can catalyze H_2_S to dissociate with intermediate laser power. In situ monitoring of time‐dependent Raman peak intensity of different phonon modes indicates a transition from WSe_2_ to WSe_2(1‐_ *_x_*_)_S_2_ *_x_* alloys, and WS_2_, as the power intensity is proportional to the number of W—S and W—Se chemical bonds (Figure 12d). Furthermore, the authors fit the time‐dependent peak intensity (*I*) from *A*_1g_ of WSe_2_ and *A*_1g_ and *E*_2g_ of WS_2_ into a double exponential equation (*I* $\propto e^{\frac{-t}{\tau 1}}+e^{\frac{-t}{\tau 2}}$) and a single exponential equation (*I* $\propto (1-e^{\frac{-t}{\tau 3}}$)), respectively, to obtain the time constant *τ* describing the photochemical conversion mechanisms (Figure 12e). With only H_2_S involved, *τ*_1_ and *τ*_2_ are 19 and 321 s, respectively. They are related to direct thermal evaporation of Se atoms that creates gaseous Se clusters and thermally activated chemical reaction between Se constituents and hydrogen in the chamber that release H_2_Se.^[^ ^82^ ^]^ Sulfur incorporation *τ*_3_ can be accelerated by introducing H_2_ into the reaction (180 s). With both H_2_S and H_2_ involved, *τ*_2_ is reduced to 162 s, indicating a rapid removal of Se constituents, thereby improving sulfur incorporation subsequently.

Laser‐assisted photonic crystallization uses amorphous precursors with predetermined molar ratios deposited by e‐beam evaporation,^[^ ^84^ ^]^ magnet‐sputtering,^[^ ^144^ ^]^ and PLD.^[^ ^145^ ^]^ Rho et al. detailed a comprehensive study on direct laser writing of MoSe_2_ by laser‐assisted selenization process. Its experimental setup includes a certain amount of Mo and Se amorphous thin films deposited on double side‐polished sapphire and a processing laser beam focusing at the Mo/Se interface to generate local heating (Figure 12f). Besides, a probe beam was used for in situ monitoring of time‐dependent laser reflectance that can, in turn, provide MoSe_2_ thickness information. The authors performed construct the theoretical relationships between laser reflectance versus amorphous Se film thickness and the resulted MoSe_2_ thickness, which is essential for obtaining desired material thickness from photonic crystallization (Figure 12g). This approach can pattern crystallized MoSe_2_ into any desired shapes, such as lines and arbitrary features with optimized laser scanning rates (Figure 12h). In addition, Raman spectra obtained from both pristine and irradiated areas indicate *α*‐phase Se amorphous film and the phonon modes of MoSe_2_ (Figure 12i), indicating laser irradiation assisted Se incorporating into Mo, transforming to the most stable H‐phase MoSe_2_. So far, laser writing for making doped or alloyed 2D TMDs is at the development stage. We believe that any approach that has been demonstrated for making standard TMDs can be easily adopted for compositional engineering because the ability to heterogeneously deposit multiple amorphous elements for specific compositions prior to laser‐assisted growth.

## 6 Conclusion

Doping and alloying in 2D materials are efficient ways to tune the optical and electronic properties, induce new crystal structures and phases, and add new functionalities. This report reviewed several practical thin‐film techniques, including CVD, MOCVD, PLD, MBE, ALD, and ion implantation for the compositional engineering of 2D semiconductor TMDs. These controlled synthesis and processing techniques demonstrated how to effectively and precisely control the type, density, and distribution of the dopants in 2D materials and adequately compensate for the limitations of bulk crystal growth methods. Also, most of the reviewed techniques are scalable for large‐area, wafer‐scale growth and processing.

Compared to the top–down processing methods, bottom‐up synthetic composition engineering through CVD_SS_, PLD, MOCVD, and MBE is a more desirable strategy to control doping and alloying in 2D materials via non‐equilibrium growth. CVD_SS_ is arguably the easiest to set up and can provide large single‐crystalline 2D TMD domains if the right recipe is established. The challenges for CVD_SS_ include the difficulty of controlling the flow of vaporized precursors, poor reproducibility, and non‐uniform domain size and material stoichiometry depending on the distance between the precursor and substrate position. The main challenge for doping and alloying 2D materials by CVD lies in the non‐synchronous evaporation of the transition metal dopants, such as Fe, Cr, Co, etc., which results in heterogeneous elemental distribution or 3D aggregations. In CVD, this presents an opportunity to use non‐equilibrium approaches to control growth kinetics by tuning the chemical potential of reactants and dopants or substrate interactions during synthesis, therefore enhancing the doping efficiency of unfavored dopants by reducing their formation energy. Pulsed layer deposition is highly versatile for doping and alloying. The thickness is controlled down to monolayer thickness by in situ laser reflectance.^[^ ^66^ ^]^ Although PLD can provide uniform 2D alloys and perform at relatively low temperatures (e.g., 400 °C),^[^ ^106^, ^107^ ^]^ the domain size, layer thickness, and crystallinity of PLD‐grown samples still need optimized. The main challenge is to reduce a high nucleation density during the initial deposition. The solutions include optimizing the power density of pulsed laser ablation, the KE (which is primarily controlled by the background gas pressure) and flux of plasma plumes, and the target preparation such as the sizes and impurity of mixed powders, and hot press conditions that control the final density of the target. Additionally, PLD systems with two or more targets are necessary to increase the flexibility for doping 2D materials or compensate for the element loss during the growth. In situ diagnostic techniques, such as absorption/reflectivity, Raman, PL, and ion mass spectroscopy need to be developed to understand the crystallinity, defects, and electronic structures under different processing conditions or environments during growth.

MOCVD provides superior controllability for tuning the compositions of TMD films. It can grow epitaxial TMDs on single‐crystalline substrates and exhibit excellent growth stability and reproducibility for a particular dopant concentration, which is essential for realizing doped TMDs in practical electronic applications. Recently, it has been used to grow tunable MoS_2(1‐_ *_x_*_)_Te_2_ *_x_* alloy films wherein Se and Te are both from MO precursors.^[^ ^146^ ^]^ New efforts for diversifying doped and alloyed samples grown by MOCVD should be spent on exploring metal or magnetic dopants and their corresponding MO precursors with a high or intermediate vapor pressure. It has been found that even with only CH‐free molecules such as W(CO)_6_ and H_2_Se involved in MOCVD of WSe_2_, the carbon impurities with a concentration of 225 ppm were still detected via STM/STS.^[^ ^147^ ^]^ While carbon impurities could raise interesting spin properties^[^ ^148^ ^]^ in 2D TMDs, it is imperative to remove them to minimize the impurity scattering and deep trap states in the devices made of TMDs. MBE can provide the cleanest synthetic crystals among all synthesis techniques because of its ultra‐high vacuum growth environment and the use of high‐purity elemental sources. Currently, MBE‐grown TMDs are still troubled by small domain sizes and polycrystallinity due to the limited surface diffusion mobility of transition metal clusters.^[^ ^68^ ^]^ Therefore, atomically flat 2D material substrates are the primary substrates to reduce diffusion barriers for adatoms in the MBE experiments. One challenge for MBE‐grown TMD alloy films is inherently from the materials themselves such as structural instability seen in Mo*_x_*V_1‐_ *_x_*Se_2_^[^ ^65^ ^]^ and WSe_2(1‐_ *_x_*_)_Te_2_ *_x_* ^[^ ^64^ ^]^ at a certain compositional ratio that results in phase separation. Nevertheless, experimentalists prefer high purity 2D TMD alloys grown by MBE in fundamental research that takes advantage of their clean band structures.^[^ ^149^ ^]^

Similar to bottom–up synthesis, top–down processes also effectively dope various elements in 2D materials. A variety of post‐synthesis implantation techniques by thermal annealing and plasma‐, electron‐, ion‐, or laser‐beam irradiation have been used to selectively dope atoms into 2D materials to form random alloys, highly ordered Janus monolayers, metastable phases, or new structures, thereby impacting the optical and electronic properties of 2D materials for electronic devices. The top–down processes' challenges can be crystal imperfection, such as defects, wrinkles, and cracks induced by dopant incorporation. For example, high‐temperature sulfurization of 2D MoSe_2_ results in surface cracking due to large tensile strains induced by S–Se exchange.^[^ ^150^ ^]^ Similarly, Se atoms implanted into 2D MoS_2_ can create wrinkles on the surface due to the stretched lattice constant caused by larger Se.^[^ ^126^ ^]^ The difficulty in releasing newly introduced strains after doping or implantation could come from the strong material‐substrate bonding that limits the lateral lattice expansion or contraction for strain release. Therefore, one needs to consider substrates for 2D crystal growth that adequately address issues raised by the post‐synthesis implantation. For instance, the frictionless surface provided by graphene and hBN^[^ ^151^ ^]^ may be substrates for doped/alloyed 2D TMDs and is also beneficial for the strain release after implanting processes. Considering ion implantation or cluster irradiation, the KE of ions must be reduced because they can penetrate implanted 2D crystals and sputter the substrate (e.g., SiO_2_ or sapphire) below, leading to material ejection that can damage 2D crystals.^[^ ^152^ ^]^

Although the direct laser writing of amorphous precursors for synthesizing pristine and doped TMDs is still in its infancy, it is promising for room‐temperature fabrication of flexible devices. The challenge is to deposit the correct amount of precursors on substrates that can be written into the desired stoichiometry and thickness on substrates with different optical absorption and thermal conductivity. The development of automated synthesis and processing instrumentation that predicts precursor thicknesses, power density for laser writing, irradiating pulse numbers, and duration in return will accelerate this emerging approach's technological readiness. Other opportunities include implantation techniques to selectively doping 2D materials to tune the degenerate or nondegenerate doping to make heterostructures for seamless contact and p–n junctions for 2D electronics. It is also important to precisely control the individual dopant atoms within 2D materials for spin electronics and quantum emitters. The critical challenges of doping 2D materials also require well‐controlled synthesis and processing with advanced in situ diagnostic techniques to fully understand the growth mechanism and the correlation between dopants and their emerging functionality. While numerous new heterogeneities and functionalities can be created in TMDs through doping and alloying,^[^ ^28^ ^]^ experimentalists should carefully evaluate each technique's desirable capabilities and the material system for exploring the specific functionalities of their interest. Therefore, controlling the doping in 2D materials provides a tremendous opportunity for future optical and electronic applications.

## Conflict of Interest

The authors declare no conflict of interest.
